# Impacts of social and economic factors on the transmission of coronavirus disease 2019 (COVID-19) in China

**DOI:** 10.1007/s00148-020-00778-2

**Published:** 2020-05-09

**Authors:** Yun Qiu, Xi Chen, Wei Shi

**Affiliations:** 1 Institute for Economic and Social Research, Jinan University, Guangzhou, Guangdong Province China; 2 Department of Health Policy and Management, Yale School of Public Health, New Haven, CT USA; 3 Department of Economics, Yale University, New Haven, CT USA

**Keywords:** 2019 novel coronavirus, Transmission, Quarantine, I18, I12, C23

## Abstract

This study models local and cross-city transmissions of the novel coronavirus in China between January 19 and February 29, 2020. We examine the role of various socioeconomic mediating factors, including public health measures that encourage social distancing in local communities. Weather characteristics 2 weeks prior are used as instrumental variables for causal inference. Stringent quarantines, city lockdowns, and local public health measures imposed in late January significantly decreased the virus transmission rate. The virus spread was contained by the middle of February. Population outflow from the outbreak source region posed a higher risk to the destination regions than other factors, including geographic proximity and similarity in economic conditions. We quantify the effects of different public health measures in reducing the number of infections through counterfactual analyses. Over 1.4 million infections and 56,000 deaths may have been avoided as a result of the national and provincial public health measures imposed in late January in China.

## Introduction

The first pneumonia case of unknown cause was found close to a seafood market in Wuhan, the capital city of Hubei province, China, on December 8, 2019. Several clusters of patients with similar pneumonia were reported through late December 2019. The pneumonia was later identified to be caused by a new coronavirus (severe acute respiratory syndrome coronavirus 2, or SARS-CoV-2) (Zhu et al. 2020), later named Coronavirus Disease 2019 (COVID-19) by the World Health Organization (WHO).^1^ While the seafood market was closed on January 1, 2020, a massive outflow of travelers during the Chinese Spring Festival travel rush (*Chunyun*) in mid-January^2^ led to the rapid spread of COVID-19 throughout China and to other countries. The first confirmed case outside Wuhan in China was reported in Shenzhen on January 19 (Li et al. 2020). As of April 5, over 1.2 million confirmed cases were reported in at least 200 countries or territories.^3^

Two fundamental strategies have been taken globally, one focused on mitigating but not necessarily stopping the virus spread and the other relying on more stringent measures to suppress and reverse the growth trajectories. While most Western countries initially implemented the former strategy, more and more of them (including most European countries and the USA) have since shifted towards the more stringent suppression strategy, and some other countries such as China, Singapore, and South Korea have adopted the latter strategy from the beginning. In particular, China has rolled out one of the most stringent public health strategies. That strategy involves city lockdowns and mandatory quarantines to ban or restrict traffic since January 23, social distance–encouraging strategies since January 28, and a centralized treatment and isolation strategy since February 2.

This study estimates how the number of daily newly confirmed COVID-19 cases in a city is influenced by the number of new COVID-19 cases in the same city, nearby cities, and Wuhan during the preceding 2 weeks using the data on confirmed COVID-19 case counts in China from January 19 to February 29. By comparing the estimates before and after February 2, we examine whether the comprehensive set of policies at the national scale delays the spread of COVID-19. Besides, we estimate the impacts of social distancing measures in reducing the transmission rate utilizing the closed management of communities and family outdoor restrictions policies that were gradually rolled out across different cities.

As COVID-19 evolves into a global pandemic and mitigating strategies are faced with growing pressure to flatten the curve of virus transmissions, more and more nations are considering implementing stringent suppression measures. Therefore, examining the factors that influence the transmission of COVID-19 and the effectiveness of the large-scale mandatory quarantine and social distancing measures in China not only adds to our understanding of the containment of COVID-19 but also provides insights into future prevention work against similar infectious diseases.

In a linear equation of the current number of new cases on the number of new cases in the past, the unobserved determinants of new infections may be serially correlated for two reasons. First, the number of people infected by a disease usually first increases, reaches a peak, and then drops. Second, there are persistent, unobservable variables, such as clusters that generate large numbers of infections, people’s living habits, and government policies. Serial correlations in errors give rise to correlations between the lagged numbers of cases and the error term, rendering the ordinary least square (OLS) estimator biased. Combining insights in Adda (2016), the existing knowledge of the incubation period of COVID-19 (World Health Organization 2020b), and data on weather conditions that affect the transmission rates of COVID-19 (Lowen and Steel 2014; Wang et al. 2020b), we construct instrumental variables for the number of new COVID-19 cases during the preceding 2 weeks. Weather characteristics in the previous third and fourth weeks do not directly affect the number of new COVID-19 cases after controlling for the number of new COVID-19 cases and weather conditions in the preceding first and second weeks. Therefore, our estimated impacts have causal interpretations and reflect population transmission rates.

Meanwhile, we estimate the mediating effects of socioeconomic factors on the transmission of COVID-19 in China. These factors include population flow out of Wuhan, the distance between cities, GDP per capita, the number of doctors, and contemporaneous weather conditions. We examine whether population flows from the origin of the COVID-19 outbreak, which is a major city and an important transportation hub in central China, can explain the spread of the virus using data on real-time travel intensity between cities that have recently become available for research. Realizing the urgency of forestalling widespread community transmissions in areas that had not seen many infections, in late January, many Chinese cities implemented public health measures that encourage social distancing. We also examine the impacts of these measures on curtailing the spread of the virus.

We find that transmission rates were lower in February than in January, and cities outside Hubei province had lower transmission rates. Preventing the transmission rates in non-Hubei cities from increasing to the level observed in late January in Hubei caused the largest reduction in the number of infections. Apart from the policies implemented nationwide, the additional social distancing policies imposed in some cities in late January further helped reduce the number of infections. By mid February, the spread of the virus was contained in China. While many socioeconomic factors moderated the spread of the virus, the actual population flow from the source posed a higher risk to destinations than other factors such as geographic proximity and similarity in economic conditions.

Our analysis contributes to the existing literature in three aspects. First, our analysis is connected to the economics and epidemiological literature on the determinants of the spread of infectious diseases and prevention of such spread. Existing studies find that reductions in population flow (Zhan et al. 2020; Zhang et al. 2020; Fang et al. 2020) and interpersonal contact from holiday school closings (Adda 2016), reactive school closures (Litvinova et al. 2019), public transportation strikes (Godzinski and Suarez Castillo 2019), strategic targeting of travelers from high-incidence locations (Milusheva 2017), and paid sick leave to keep contagious workers at home (Barmby and Larguem 2009; Pichler and Ziebarth 2017) can mitigate the prevalence of disease transmissions. In addition, studies show viruses spread faster during economic booms (Adda 2016), increases in employment are associated with increased incidence of influenza (Markowitz et al. 2019), and growth in trade can significantly increase the spread of influenza (Adda 2016) and HIV (Oster 2012). Vaccination (Maurer 2009; White 2019) and sunlight exposure (Slusky and Zeckhauser 2018) are also found effective in reducing the spread of influenza.

Second, our paper adds to the epidemiological studies on the basic reproduction number (*R*_0_) of COVID-19, i.e., the average number of cases directly generated by one case in a population where all individuals are susceptible to infection. Given the short time period since the beginning of the COVID-19 outbreak, research is urgently needed to assess the dynamics of transmissions and the implications for how the COVID-19 outbreak will evolve (Wu et al. 2020b, 2020c). Liu et al. (2020) identify 12 studies that estimated the basic reproductive number in the wide range of 1.4 to 6.5 (with a mean of 3.28 and a median of 2.79) for Wuhan, Hubei, China, or overseas during January 1 through January 28, 2020.^4^ Our *R*_0_ estimate relies on spatially disaggregated data during an extended period (until February 29, 2020) to mitigate potential biases, and the instrumental variable approach we use isolates the causal effect of virus transmissions and imposes fewer restrictions on the relationship between the unobserved determinants of new cases and the number of cases in the past. Simultaneously considering a more comprehensive set of factors in the model that may influence virus spread, we find that one case generates 2.992 more cases within 2 weeks (1.876 if cities in Hubei province are excluded) in the sub-sample from January 19 to February 1. In the sub-sample from February 2 to February 29, the transmission rates fall to 1.243 (0.614 excluding Hubei province). Our estimate of *R*_0_ for the period in late January 2020 that overlaps with existing studies falls well within the range of the estimated *R*_0_ in the emerging COVID-19 literature (Liu et al. 2020).

Third, our study contributes to the assessments of public health measures aiming at reducing virus transmissions and mortality. Through a set of policy simulations, we report initial evidence on the number of avoided infections through the end of February 2020 for cities outside Hubei province. Specifically, the stringent health policies at the national and provincial levels reduced the transmission rate and resulted in 1,408,479 (95% CI, 815,585 to 2,001,373) fewer infections and potentially 56,339 fewer deaths.^5^ In contrast, the effects of the Wuhan lockdown and local non-pharmaceutical interventions (NPIs) are considerably smaller. As a result of the Wuhan lockdown, closed management of communities, and family outdoor restrictions, 31,071 (95% CI, 8296 to 53,845), 3803 (95% CI, 1142 to 6465), and 2703 (95% CI, 654 to 4751) fewer cases were avoided, respectively. These three policies may respectively avoid 1,243 deaths, 152 deaths, and 108 deaths. Making some additional assumptions, such as the value of statistical life and lost productive time, these estimates may provide the basis for more rigorous cost-benefit analysis regarding relevant public health measures.

This paper is organized as follows. Section 2 introduces the empirical model. Section 3 discusses our data and the construction of key variables. Section 4 presents the results. Section 5 documents the public health measures implemented in China, whose impacts are quantified in a series of counterfactual exercises. Section 6 concludes. The Appendix contains additional details on the instrumental variables, data quality, and the computation of counterfactuals.

## Empirical model

Our analysis sample includes 304 prefecture-level cities in China. We exclude Wuhan, the capital city of Hubei province, from our analysis for two reasons. First, the epidemic patterns in Wuhan are significantly different from those in other cities. Some confirmed cases in Wuhan contracted the virus through direct exposure to Huanan Seafood Wholesale Market, which is the most probable origin of the virus^6^. In other cities, infections arise from human-to-human transmissions. Second, COVID-19 cases were still pneumonia of previously unknown virus infections in people’s perception until early January so that Wuhan’s health care system became overwhelmed as the number of new confirmed cases increased exponentially since mid-January. This may have caused severe delay and measurement errors in the number of cases reported in Wuhan, and to a lesser extent, in other cities in Hubei province. To alleviate this concern, we also conduct analyses excluding all cities in Hubei province from our sample.

To model the spread of the virus, we consider within-city spread and between-city transmissions simultaneously (Adda 2016). Our starting point is

$$ y_{ct}=\sum\limits_{s=1}^{14}\alpha_{\text{within},s}y_{c,t-s}+\sum\limits_{s=1}^{14}\alpha_{\text{between},s}\sum\limits_{r\neq c}d_{cr}^{-1}y_{r,t-s}+\sum\limits_{s=1}^{14}\rho_{s}z_{t-s}+x_{ct}\upbeta+\epsilon_{ct}, $$

where *c* is a city other than Wuhan, and *y*_*ct*_ is the number of new confirmed cases of COVID-19 in city *c* on date *t*. Regarding between-city transmissions, *d*_*cr*_ is the log of the distance between cities *c* and *r*, and ${\sum }_{r\neq c}d_{cr}^{-1}y_{rt}$ is the inverse distance weighted sum of new infections in other cities. Considering that COVID-19 epidemic originated from one city (Wuhan) and that most of the early cases outside Wuhan can be traced to previous contacts with persons in Wuhan, we also include the number of new confirmed cases in Wuhan (*z*_*t*_) to model how the virus spreads to other cities from its source. We may include lagged *y*_*ct*_, *y*_*rt*_, and *z*_*t*_ up to 14 days based on the estimates of the durations of the infectious period and the incubation period in the literature^7^. *x*_*ct*_ includes contemporaneous weather controls, city, and day fixed effects^8^. *𝜖*_*ct*_ is the error term. Standard errors are clustered by province.

To make it easier to interpret the coefficients, we assume that the transmission dynamics (*α*_within,*s*_, *α*_between,*s*_, *ρ*_*s*_) are the same within *s* = 1,⋯ ,7 and *s* = 8,⋯ ,14, respectively, but can be different across weeks. Specifically, we take averages of lagged *y*_*ct*_, *y*_*rt*_, and *z*_*t*_ by week, as $\bar {y}_{ct}^{\tau }=\frac {1}{7}{\sum }_{s=1}^{7}y_{ct-7\left (\tau -1\right )-s}$, $\bar {y}_{rt}^{\tau }=\frac {1}{7}{\sum }_{s=1}^{7}y_{rt-7\left (\tau -1\right )-s}$, and $\bar {z}_{t}^{\tau }=\frac {1}{7}{\sum }_{s=1}^{7}z_{t-7\left (\tau -1\right )-s}$, in which *τ* denotes the preceding first or second week. Our main model is

1 $$ y_{ct}=\sum\limits_{\tau=1}^{2}\alpha_{\text{within},\tau}\bar{y}_{ct}^{\tau}+\sum\limits_{\tau=1}^{2}\alpha_{\text{between},\tau}\sum\limits_{r\neq c}d_{cr}^{-1}\bar{y}_{rt}^{\tau}+\sum\limits_{\tau=1}^{2}\rho_{\tau}\bar{z}_{t}^{\tau}+x_{ct}\upbeta+\epsilon_{ct}.\quad\textbf{Model A} $$

We also consider more parsimonious model specifications, such as the model that only considers within-city transmissions,

2 $$ y_{ct}=\sum\limits_{\tau=1}^{2}\alpha_{\text{within},\tau}\bar{y}_{ct}^{\tau}+x_{ct}\upbeta+\epsilon_{ct}, $$

and the model where the time lagged variables are averages over the preceding 2 weeks,

$$ y_{ct}=\alpha_{\text{within}}\frac{1}{14}\sum\limits_{s=1}^{14}y_{c,t-s}+\alpha_{\text{between}}\frac{1}{14}\sum\limits_{s=1}^{14} \sum\limits_{r\neq c}d_{cr}^{-1}y_{r,t-s}+\rho\frac{1}{14}\sum\limits_{s=1}^{14}z_{t-s}+x_{ct}\upbeta+\epsilon_{ct}.\quad\textbf{Model B} $$

There are several reasons that $\bar {y}_{ct}^{\tau }$, $\bar {y}_{rt}^{\tau }$, and $\bar {z}_{t}^{\tau }$ may be correlated with the error term *𝜖*_*ct*_. The unobserved determinants of new infections such as local residents’ and government’s preparedness are likely correlated over time, which causes correlations between the error term and the lagged dependent variables. As noted by the World Health Organization (2020b), most cases that were locally generated outside Hubei occurred in households or clusters. The fact that big clusters give rise to a large number of cases within a short period of time may still be compatible with a general low rate of community transmissions, especially when measures such as social distancing are implemented. Therefore, the coefficients are estimated by two-stage least squares in order to obtain consistent estimates on the transmission rates in the population.

**Fig. 1:**
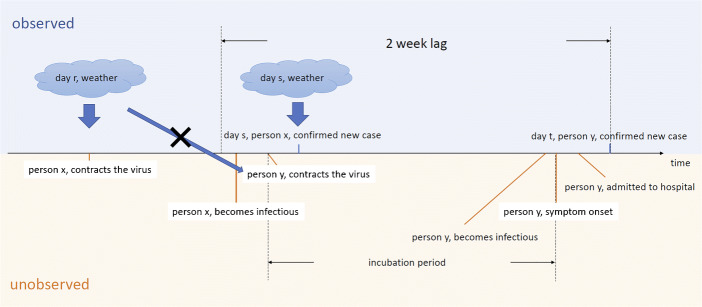
Timeline of key variables

In Eq. 2, the instrumental variables include averages of daily maximum temperature, total precipitation, average wind speed, and the interaction between precipitation and wind speed, for city *c* in the preceding third and fourth weeks. Detailed discussion of the selection of weather characteristics as instruments is in Section 3.2. The timeline of key variables are displayed in Fig. 1. The primary assumption on the instrumental variables is that weather conditions before 2 weeks do not affect the likelihood that a person susceptible to the virus contracts the disease, conditional on weather conditions and the number of infectious people within the 2-week window. On the other hand, they affect the number of other persons who have become infectious within the 2-week window, because they may have contracted the virus earlier than 2 weeks. These weather variables are exogenous to the error term and affect the spread of the virus, which have been used by Adda (2016) to instrument flu infections^9^.

Another objective of this paper is to quantify the effect of various socioeconomic factors in mediating the transmission rates of the virus, which may identify potential behavioral and socioeconomic risk factors for infections. For within-city transmissions, we consider the effects of local public health measures (see Section 5 for details) and the mediating effects of population density, level of economic development, number of doctors, and environmental factors such as temperature, wind, and precipitation. For between-city transmissions, apart from proximity measures based on geographic distance, we also consider similarity in population density and the level of economic development. To measure the spread of the virus from Wuhan, we also include the number of people traveling from Wuhan. The full empirical model is as follows:

3 $$ \begin{array}{@{}rcl@{}} y_{ct}&= & \sum\limits_{\tau=1}^{2}{\sum}_{k=1}^{K_{\text{within}}}\alpha_{\text{within},\tau}^{k}\bar{h}_{ct}^{k\tau}\bar{y}_{ct}^{\tau}+\sum\limits_{\tau=1}^{2}{\sum}_{k=1}^{K_{\text{between}}}{\sum}_{r\neq c}\alpha_{\text{between},\tau}^{k}\bar{m}_{crt}^{k\tau}\bar{y}_{rt}^{\tau}+\sum\limits_{\tau=1}^{2}{\sum}_{k=1}^{K_{\text{Wuhan}}}\rho_{\tau}^{k}\bar{m}_{c,\text{Wuhan},t}^{k\tau}\bar{z}_{t}^{\tau}\\ && + x_{ct}\upbeta+\epsilon_{ct}, \end{array} $$

where $\bar {h}_{ct}^{k\tau }$ includes dummies for local public health measures and the mediating factors for local transmissions. $\bar {m}_{crt}^{k\tau }$ and $\bar {m}_{c,\text {Wuhan},t}^{k\tau }$ are the mediating factors for between-city transmissions and imported cases from Wuhan.

## Data

### Variables

**Fig. 2:**
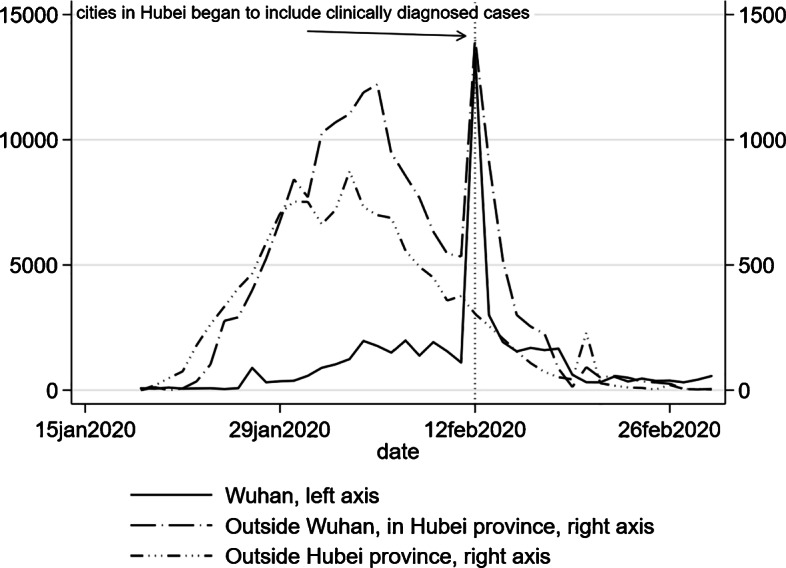
Number of daily new confirmed cases of COVID-19 in mainland China

January 19, 2020, is the first day that COVID-19 cases were reported outside of Wuhan, so we collect the daily number of new cases of COVID-19 for 305 cities from January 19 to February 29. All these data are reported by 32 provincial-level Health Commissions in China^10^. Figure 2 shows the time patterns of daily confirmed new cases in Wuhan, in Hubei province outside Wuhan, and in non-Hubei provinces of mainland China. Because Hubei province started to include clinically diagnosed cases into new confirmed cases on February 12, we notice a spike in the number of new cases in Wuhan and other cities in Hubei province on this day (Fig. 2). The common effects of such changes in case definitions on other cities can be absorbed by time fixed effects. As robustness checks, we re-estimate models A and B without the cities in Hubei province. In addition, since the number of clinically diagnosed cases at the city level was reported for the days of February 12, 13, and 14, we recalculated the daily number of new cases for the 3 days by removing the clinically diagnosed cases from our data and re-estimate models A and B. Our main findings still hold (Appendix B).

Regarding the explanatory variables, we calculate the number of new cases of COVID-19 in the preceding first and second weeks for each city on each day. To estimate the impacts of new COVID-19 cases in other cities, we first calculate the geographic distance between a city and all other cities using the latitudes and longitudes of the centroids of each city and then calculate the weighted sum of the number of COVID-19 new cases in all other cities using the inverse of log distance between a city and each of the other cities as the weight.

**Fig. 3:**
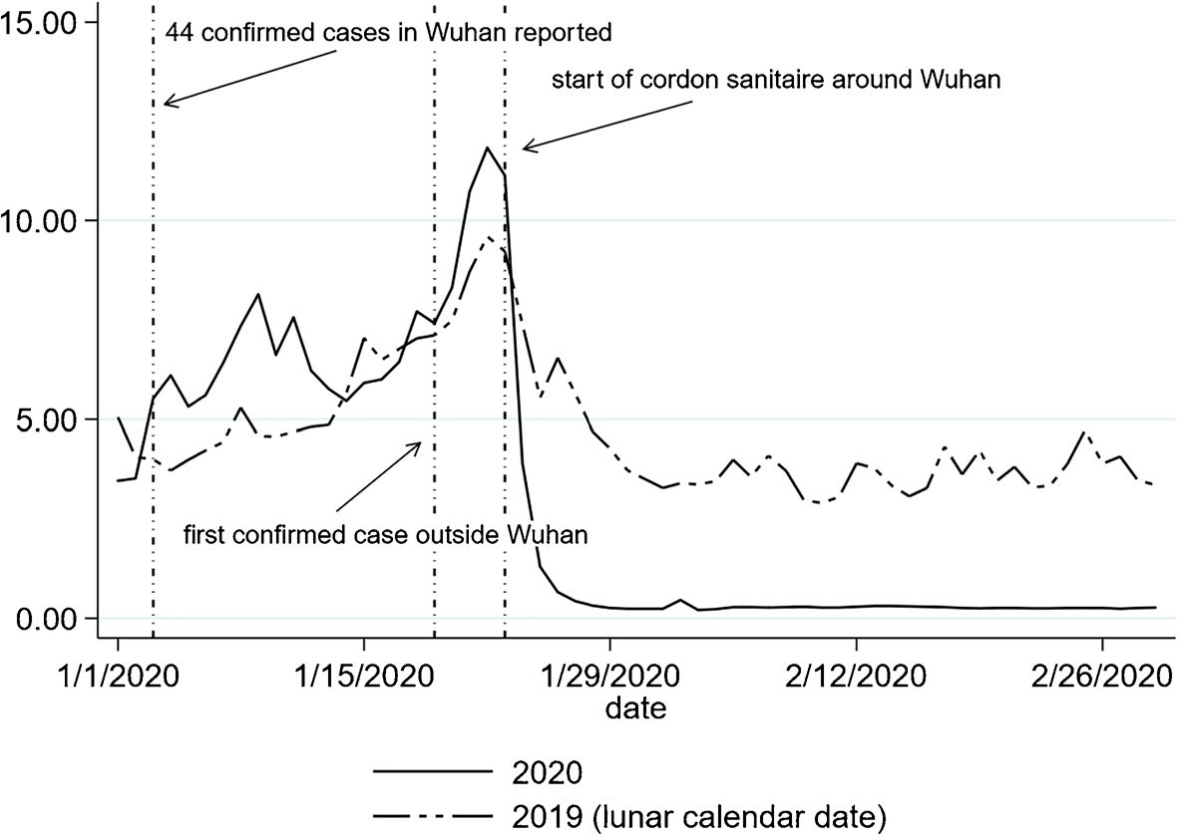
Baidu index of population flow from Wuhan

**Fig. 4:**
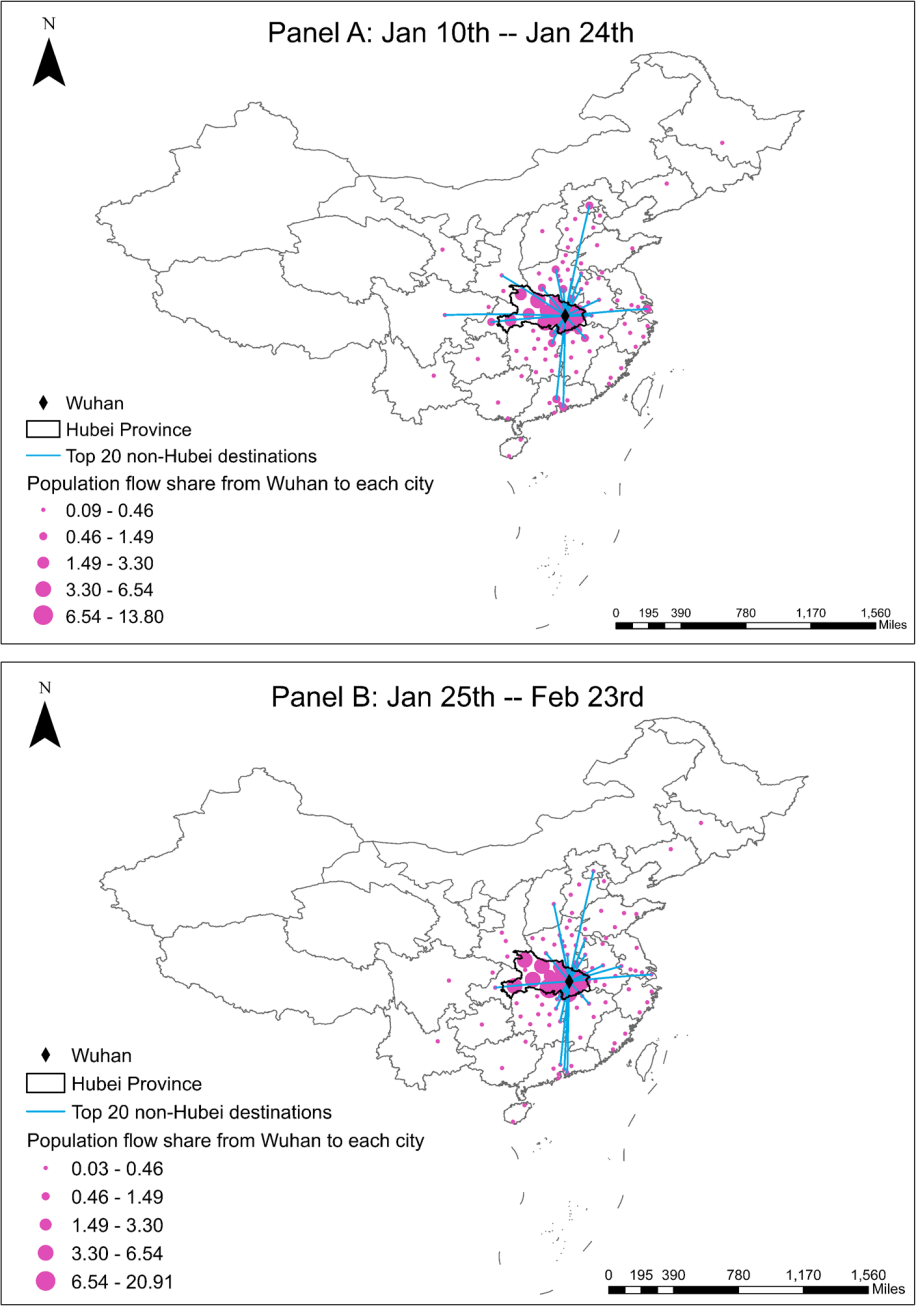
Destination shares in population flow from Wuhan

Since the COVID-19 outbreak started from Wuhan, we also calculate the weighted number of COVID-19 new cases in Wuhan using the inverse of log distance as the weight. Furthermore, to explore the mediating impact of population flow from Wuhan, we collect the daily population flow index from Baidu that proxies for the total intensity of migration from Wuhan to other cities^11^. Figure 3 plots the Baidu index of population flow out of Wuhan and compares its values this year with those in 2019. We then interact the flow index with the share that a destination city takes (Fig. 4) to construct a measure on the population flow from Wuhan to a destination city. Other mediating variables include population density, GDP per capita, and the number of doctors at the city level, which we collect from the most recent China city statistical yearbook. Table 1 presents the summary statistics of these variables. On average, GDP per capita and population density are larger in cities outside Hubei province than those in Hubei. Compared with cities in Hubei province, cities outside Hubei have more doctors.

**Table 1:** Summary statistics

Variable	*N*	Mean	Std dev.	Min.	Median	Max.
**Non Hubei cities**						
*City characteristics*						
GDP per capita, 10,000RMB	288	5.225	3.025	1.141	4.327	21.549
Population density, per km^2^	288	428.881	374.138	9.049	327.115	3444.092
# of doctors, 10,000	288	1.086	1.138	0.030	0.805	10.938
*Time varying variables, Jan 19–Feb 1*						
Daily # of new confirmed cases	4032	1.303	3.608	0.000	0.000	60.000
Weekly average max. temperature, ^∘^C	4032	8.520	8.525	− 18.468	7.932	29.833
Weekly average precipitation, mm	4032	0.238	0.558	0.000	0.033	5.570
Weekly average wind speed, m/s	4032	2.209	0.842	0.816	2.014	6.386
*Time varying variables, Feb 1–Feb 29*						
Daily # of new confirmed cases	8064	0.927	3.461	0.000	0.000	201.000
Weekly average max. temperature, ^∘^*C*	8064	11.909	7.983	− 18.032	12.814	28.791
Weekly average precipitation, mm	8064	0.193	0.491	0.000	0.027	5.432
Weekly average wind speed, m/s	8064	2.461	0.913	0.654	2.352	7.129
**Cities in Hubei province, excluding Wuhan**						
*City characteristics*						
GDP per capita, 10,000RMB	16	4.932	1.990	2.389	4.306	8.998
Population density, per km^2^	16	416.501	220.834	24.409	438.820	846.263
# of doctors, 10,000	16	0.698	0.436	0.017	0.702	1.393
*Time varying variables, Jan 19–Feb 1*						
Daily # of new confirmed cases	224	22.165	35.555	0.000	7.000	276.000
Weekly average max. temperature, ^∘^C	224	8.709	1.602	1.278	8.905	10.889
Weekly average precipitation, mm	224	0.261	0.313	0.000	0.160	1.633
Weekly average wind speed, m/s	224	1.970	0.600	0.893	1.975	3.439
*Time varying variables, Feb 1–Feb 29*						
Daily # of new confirmed cases	448	28.871	51.793	0.000	8.000	424.000
Weekly average max. temperature, ^∘^C	448	14.569	2.985	1.452	14.448	23.413
Weekly average precipitation, mm	448	0.201	0.233	0.000	0.133	1.535
Weekly average wind speed, m/s	448	2.063	0.648	0.705	2.070	4.174

Variables of the city characteristics are obtained from City Statistical Yearbooks. Time varying variables are observed daily for each city. Weekly average weather variables are averages over the preceding week

We rely on meteorological data to construct instrumental variables for the endogenous variables. The National Oceanic and Atmospheric Administration (NOAA) provides average, maximum, and minimum temperatures, air pressure, average and maximum wind speeds, precipitation, snowfall amount, and dew point for 362 weather stations at the daily level in China. To merge the meteorological variables with the number of new cases of COVID-19, we first calculate daily weather variables for each city on each day from 2019 December to 2020 February from station-level weather records following the inverse distance weighting method. Specifically, for each city, we draw a circle of 100 km from the city’s centroid and calculate the weighted average daily weather variables using stations within the 100-km circle^12^. We use the inverse of the distance between the city’s centroid and each station as the weight. Second, we match the daily weather variables to the number of new cases of COVID-19 based on city name and date.

### Selection of instrumental variables

The transmission rate of COVID-19 may be affected by many environmental factors. Human-to-human transmission of COVID-19 is mostly through droplets and contacts (National Health Commission of the PRC 2020). Weather conditions such as rainfall, wind speed, and temperature may shape infections via their influences on social activities and virus transmissions. For instance, increased precipitation results in higher humidity, which may weaken virus transmissions (Lowen and Steel 2014). The virus may survive longer with lower temperature (Wang et al. 2020b; Puhani 2020). Greater wind speed and therefore ventilated air may decrease virus transmissions. In addition, increased rainfall and lower temperature may also reduce social activities. Newly confirmed COVID-19 cases typically arise from contracting the virus within 2 weeks in the past (e.g., World Health Organization 2020b). The extent of human-to-human transmission is determined by the number of people who have already contracted the virus and the environmental conditions within the next 2 weeks. Conditional on the number of people who are infectious and environmental conditions in the previous first and second weeks, it is plausible that weather conditions further in the past, i.e., in the previous third and fourth weeks, should not directly affect the number of current new cases. Based on the existing literature, we select weather characteristics as the instrumental variables, which include daily maximum temperature, precipitation, wind speed, and the interaction between precipitation and wind speed.

We then regress the endogenous variables on the instrumental variables, contemporaneous weather controls, city, date, and city by week fixed effects. Table 2 shows that F-tests on the coefficients of the instrumental variables all reject joint insignificance, which confirms that overall the selected instrumental variables are not weak. The coefficients of the first stage regressions are reported in Table 9 in the appendix.

**Table 2:** First stage results

		Jan 19–Feb 29	Jan 19–Feb 1	Feb 2–Feb 29
*Own city*				
Average # new cases, 1-week lag	*F* stat	11.41	4.02	17.28
	*p* value	0.0000	0.0000	0.0000
Average # new cases, 2-week lag	*F* stat	8.46	5.66	10.25
	*p* value	0.0000	0.0000	0.0000
Average # new cases, previous 14 days	*F* stat	18.37	7.72	21.69
	*p* value	0.0000	0.0000	0.0000
*Other cities, inverse distance weighted*				
Average # new cases, 1-week lag	*F* stat	19.10	36.29	17.58
	*p* value	0.0000	0.0000	0.0000
Average # new cases, 2-week lag	*F* stat	36.32	19.94	37.31
	*p* value	0.0000	0.0000	0.0000
Average # new cases, previous 14 days	*F* stat	47.08	33.45	46.22
	*p* value	0.0000	0.0000	0.0000

This table reports the *F*-tests on the joint significance of the coefficients on the instrumental variables (IV) that are excluded from the estimation equations. Our IV include weekly averages of daily maximum temperature, precipitation, wind speed, and the interaction between precipitation and wind speed, during the preceding third and fourth weeks, and the averages of these variables in other cities weighted by the inverse of log distance. For each *F* statistic, the variable in the corresponding row is the dependent variable, and the time window in the corresponding column indicates the time span of the sample. Each regression also includes 1- and 2-week lags of these weather variables, weekly averages of new infections in the preceding first and second weeks in Wuhan which are interacted with the inverse log distance or the population flow, and city, date and city by week fixed effects. Coefficients on the instrumental variables for the full sample are reported in Table 15 in the appendix

We also need additional weather variables to instrument the adoption of public health measures at the city level. Since there is no theoretical guidance from the existing literature, we implement the Cluster-Lasso method of Belloni et al. (2016) and Ahrens et al. (2019) to select weather characteristics that have good predictive power. Details are displayed in Appendix A.

## Results

Our sample starts from January 19, when the first COVID-19 case was reported outside Wuhan. The sample spans 6 weeks in total and ends on February 29. We divide the whole sample into two sub-samples (January 19 to February 1, and February 2 to February 29) and estimate the model using the whole sample and two sub-samples, respectively. In the first 2 weeks, COVID-19 infections quickly spread throughout China with every province reporting at least one confirmed case, and the number of cases also increased at an increasing speed (Fig. 2). It is also during these 2 weeks that the Chinese government took actions swiftly to curtail the virus transmission. On January 20, COVID-19 was classified as a class B statutory infectious disease and treated as a class A statutory infectious disease. The city of Wuhan was placed under lockdown on January 23; roads were closed, and residents were not allowed to leave the city. Many other cities also imposed public policies ranging from canceling public events and stopping public transportation to limiting how often residents could leave home. By comparing the dynamics of virus transmissions in these two sub-samples, we can infer the effectiveness of these public health measures.

In this section, we will mostly rely on model A to interpret the results, which estimates the effects of the average number of new cases in the preceding first and second week, respectively, and therefore enables us to examine the transmission dynamics at different time lags. As a robustness check, we also consider a simpler lag structure to describe the transmission dynamics. In model B, we estimate the effects of the average number of new cases in the past 14 days instead of using two separate lag variables.

### Within-city transmission

Table 3 reports the estimation results of the OLS and IV regressions of Eq. 2, in which only within-city transmission is considered. After controlling for time-invariant city fixed effects and time effects that are common to all cities, on average, one new infection leads to 1.142 more cases in the next week, but 0.824 fewer cases 1 week later. The negative effect can be attributed to the fact that both local authorities and residents would have taken more protective measures in response to a higher perceived risk of contracting the virus given more time. Information disclosure on newly confirmed cases at the daily level by official media and information dissemination on social media throughout China may have promoted more timely actions by the public, resulting in slower virus transmissions. We then compare the transmission rates in different time windows. In the first sub-sample, one new infection leads to 2.135 more cases within a week, implying a fast growth in the number of cases. However, in the second sub-sample, the effect decreases to 1.077, suggesting that public health measures imposed in late January were effective in limiting a further spread of the virus. Similar patterns are also observed in model B.

**Table 3:** Within-city transmission of COVID-19

	Jan 19–Feb 29		Jan 19–Feb 1		Feb 2–Feb 29	
	(1)	(2)	(3)	(4)	(5)	(6)
	OLS	IV	OLS	IV	OLS	IV
*All cities excluding Wuhan*						
Model A: lagged variables are averages over the preceding first and second week separately						
Average # of new cases	0.873***	1.142***	1.692***	2.135***	0.768***	1.077***
1-week lag	(0.00949)	(0.0345)	(0.0312)	(0.0549)	(0.0120)	(0.0203)
Average # of new cases	− 0.415***	− 0.824***	0.860	− 6.050***	− 0.408***	− 0.796***
2-week lag	(0.00993)	(0.0432)	(2.131)	(2.314)	(0.00695)	(0.0546)
Model B: lagged variables are averages over the preceding 2 weeks						
Average # of new case	0.474***	0.720***	3.310***	3.860***	0.494***	1.284***
Previous 14 days	(0.0327)	(0.143)	(0.223)	(0.114)	(0.00859)	(0.107)
Observations	12,768	12,768	4256	4256	8512	8512
Number of cities	304	304	304	304	304	304
Weather controls	Yes	Yes	Yes	Yes	Yes	Yes
City FE	Yes	Yes	Yes	Yes	Yes	Yes
Date FE	Yes	Yes	Yes	Yes	Yes	Yes
*All cities excluding cities in Hubei Province*						
Model A: lagged variables are averages over the preceding first and second week separately						
Average # of new cases	0.725***	1.113***	1.050***	1.483***	0.620***	0.903***
1-week lag	(0.141)	(0.0802)	(0.0828)	(0.205)	(0.166)	(0.0349)
Average # of new cases	− 0.394***	− 0.572***	0.108	− 3.664	− 0.228***	− 0.341***
2-week lag	(0.0628)	(0.107)	(0.675)	(2.481)	(0.0456)	(0.121)
Model B: lagged variables are averages over the preceding 2 weeks						
Average # of new cases	0.357***	0.631***	1.899***	2.376***	0.493***	0.745***
Previous 14 days	(0.0479)	(0.208)	(0.250)	(0.346)	(0.122)	(0.147)
Observations	12,096	12,096	4032	4032	8064	8064
Number of cities	288	288	288	288	288	288
Weather controls	Yes	Yes	Yes	Yes	Yes	Yes
City FE	Yes	Yes	Yes	Yes	Yes	Yes
Date FE	Yes	Yes	Yes	Yes	Yes	Yes

The dependent variable is the number of daily new cases. The endogenous explanatory variables include the average numbers of new confirmed cases in the own city in the preceding first and second weeks (model A) and the average number in the preceding 14 days (model B). Weekly averages of daily maximum temperature, precipitation, wind speed, the interaction between precipitation and wind speed, and the inverse log distance weighted sum of each of these variables in other cities, during the preceding third and fourth weeks, are used as instrumental variables in the IV regressions. Weather controls include contemporaneous weather variables in the preceding first and second weeks. Standard errors in parentheses are clustered by provinces. *** *p* < 0.01, ** *p* < 0.05, * *p* < 0.1

Many cases were also reported in other cities in Hubei province apart from Wuhan, where six of them reported over 1000 cumulative cases by February 15^13^. Their overstretched health care system exacerbates the concern over delayed reporting of confirmed cases in these cities. To mitigate the effect of such potential measurement errors on our estimates, we re-estimate (2) excluding all cities in Hubei province. The bottom panel of Table 3 reports these estimates. Comparing the IV estimates in columns (4) and (6) between the upper and lower panels, we find that the transmission rates are lower in cities outside Hubei. In the January 19–February 1 sub-sample, one new case leads to 1.483 more cases in the following week, and this is reduced to 0.903 in the February 2–February 29 sub-sample. We also find a similar pattern when comparing the estimates from model B.

### Between-city transmission

People may contract the virus from interaction with the infected people who live in the same city or other cities. In Eq. 1, we consider the effects of the number of new infections in other cities and in the epicenter of the epidemic (Wuhan), respectively, using inverse log distance as weights. In addition, geographic proximity may not fully describe the level of social interactions between residents in Wuhan and other cities since the lockdown in Wuhan on January 23 significantly reduced the population flow from Wuhan to other cities. To alleviate this concern, we also use a measure of the size of population flow from Wuhan to a destination city, which is constructed by multiplying the daily migration index on the population flow out of Wuhan (Fig 3) with the share of the flow that a destination city receives provided by Baidu (Fig. 4). For days before January 25, we use the average destination shares between January 10 and January 24. For days on or after January 24, we use the average destination shares between January 25 and February 23^14^.

Table 4 reports the estimates from IV regressions of Eq. 1, and Table 5 reports the results from the same regressions excluding Hubei province. Column (4) of Table 4 indicates that in the first sub-sample, one new case leads to 2.456 more cases within 1 week, and the effect is not statistically significant between 1 and 2 weeks. Column (6) suggests that in the second sub-sample, one new case leads to 1.127 more cases within 1 week, and the effect is not statistically significant between 1 and 2 weeks. The comparison of the coefficients on own city between different sub-samples indicates that the responses of the government and the public have effectively decreased the risk of additional infections. Comparing Table 4 with Table 3, we find that although the number of new cases in the preceding second week turns insignificant and smaller in magnitude, coefficients on the number of new cases in the preceding first week are not sensitive to the inclusion of terms on between-city transmissions.

**Table 4:** Within- and between-city rransmission of COVID-19

	Jan 19–Feb 29		Jan 19–Feb 1		Feb 2–Feb 29	
	(1)	(2)	(3)	(4)	(5)	(6)
	OLS	IV	OLS	IV	OLS	IV
Model A: lagged variables are averages over the preceding first and second week separately						
Average # of new cases, 1-week lag						
Own city	0.862***	1.387***	0.939***	2.456***	0.786***	1.127***
	(0.0123)	(0.122)	(0.102)	(0.638)	(0.0196)	(0.0686)
Other cities	0.00266	− 0.0248	0.0889	0.0412	− 0.00316	− 0.0212
*wt. = inv. dist.*	(0.00172)	(0.0208)	(0.0714)	(0.0787)	(0.00227)	(0.0137)
Wuhan	− 0.0141	0.0303	− 0.879	− 0.957	− 0.00788	0.0236
*wt. = inv. dist.*	(0.0115)	(0.0318)	(0.745)	(0.955)	(0.00782)	(0.0200)
Wuhan	3.74e-05	0.00151***	0.00462***	0.00471***	− 0.00211***	− 0.00238**
*wt. = pop. flow*	(0.000163)	(0.000391)	(0.000326)	(0.000696)	(4.01e-05)	(0.00113)
Average # of new cases, 2-week lag						
Own city	− 0.425***	− 0.795***	2.558	− 1.633	− 0.205***	− 0.171
	(0.0318)	(0.0643)	(2.350)	(2.951)	(0.0491)	(0.224)
Other cities	− 0.00451**	− 0.00766	− 0.361	− 0.0404	− 0.00912**	− 0.0230
*wt. = inv. dist.*	(0.00213)	(0.00814)	(0.371)	(0.496)	(0.00426)	(0.0194)
Wuhan	− 0.0410*	0.0438	3.053	3.031	− 0.0603	− 0.00725
*wt. = inv. dist.*	(0.0240)	(0.0286)	(2.834)	(3.559)	(0.0384)	(0.0137)
Wuhan	0.00261***	0.00333***	0.00711***	− 0.00632	0.00167**	0.00368***
*wt. = pop. flow*	(0.000290)	(0.000165)	(0.00213)	(0.00741)	(0.000626)	(0.000576)
Model B: lagged variables are averages over the preceding 2 weeks						
Own city	0.425***	1.195***	1.564***	2.992***	0.615***	1.243***
	(0.0771)	(0.160)	(0.174)	(0.892)	(0.0544)	(0.115)
Other cities	− 0.00901	− 0.0958**	0.0414	0.0704	− 0.0286***	− 0.0821***
*wt. = inv. dist.*	(0.00641)	(0.0428)	(0.0305)	(0.0523)	(0.0101)	(0.0246)
Wuhan	− 0.198*	− 0.0687**	− 0.309	− 0.608	− 0.234*	− 0.144
*wt. = inv. dist.*	(0.104)	(0.0268)	(0.251)	(0.460)	(0.121)	(0.0994)
Wuhan	0.00770***	0.00487***	0.00779***	0.00316	0.00829***	0.00772***
*wt. = pop. flow*	(0.000121)	(0.000706)	(0.000518)	(0.00276)	(0.000367)	(0.000517)
Observations	12,768	12,768	4256	4256	8512	8512
Number of cities	304	304	304	304	304	304
Weather controls	Yes	Yes	Yes	Yes	Yes	Yes
City FE	Yes	Yes	Yes	Yes	Yes	Yes
Date FE	Yes	Yes	Yes	Yes	Yes	Yes

The dependent variable is the number of daily new cases. The endogenous explanatory variables include the average numbers of new confirmed cases in the own city and nearby cities in the preceding first and second weeks (model A) and averages in the preceding 14 days (model B). Weekly averages of daily maximum temperature, precipitation, wind speed, the interaction between precipitation and wind speed, and the inverse log distance weighted sum of these variables in other cities, during the preceding third and fourth weeks, are used as instrumental variables in the IV regressions. Weather controls include contemporaneous weather variables in the preceding first and second weeks. Standard errors in parentheses are clustered by provinces. *** *p* < 0.01, ** *p* < 0.05, * *p* < 0.1

**Table 5:** Within- and between-city transmission of COVID-19, excluding cities in Hubei Province

	Jan 19–Feb 29		Jan 19–Feb 1		Feb 2–Feb 29	
	(1)	(2)	(3)	(4)	(5)	(6)
	OLS	IV	OLS	IV	OLS	IV
Model A: lagged variables are averages over the preceding first and second week separately						
Average # of new cases, 1-week lag						
Own city	0.656***	1.117***	0.792***	1.194***	0.567***	0.899***
	(0.153)	(0.112)	(0.0862)	(0.302)	(0.172)	(0.0924)
Other cities	0.00114	− 0.00213	− 0.0160	− 0.0734	0.000221	− 0.00526**
*wt. = inv. dist.*	(0.000741)	(0.00367)	(0.0212)	(0.0803)	(0.000626)	(0.00244)
Wuhan	− 0.000482	0.00420	0.104	0.233	5.89e-05	0.00769**
*wt. = inv. dist.*	(0.00173)	(0.00649)	(0.128)	(0.156)	(0.00194)	(0.00379)
Wuhan	0.00668***	0.00616***	0.00641***	0.00375	− 0.000251	0.00390
*wt. = pop. flow*	(0.00159)	(0.00194)	(0.00202)	(0.00256)	(0.00245)	(0.00393)
Average # of new cases, 2-week lag						
Own city	− 0.350***	− 0.580***	0.230	− 1.541	− 0.157**	− 0.250**
	(0.0667)	(0.109)	(0.572)	(1.448)	(0.0636)	(0.119)
Other cities	− 0.000869	0.00139	0.172	0.584	− 0.00266*	− 0.00399
*wt. = inv. dist.*	(0.00102)	(0.00311)	(0.122)	(0.595)	(0.00154)	(0.00276)
Wuhan	− 0.00461	0.000894	− 0.447	− 0.970	− 0.00456	0.00478*
*wt. = inv. dist.*	(0.00304)	(0.00592)	(0.829)	(0.808)	(0.00368)	(0.00280)
Wuhan	0.00803***	0.00203	0.00973***	0.00734	0.00759***	0.00466***
*wt. = pop. flow*	(0.00201)	(0.00192)	(0.00317)	(0.00680)	(0.00177)	(0.00140)
Model B: lagged variables are averages over the preceding 2 weeks						
Own city	0.242***	0.654***	1.407***	1.876***	0.406***	0.614***
	(0.0535)	(0.195)	(0.215)	(0.376)	(0.118)	(0.129)
Other cities	0.000309	− 0.00315	0.00608	0.0194	− 0.00224	− 0.00568
*wt. = inv. dist.*	(0.00142)	(0.00745)	(0.0188)	(0.0300)	(0.00204)	(0.00529)
Wuhan	− 0.0133**	− 0.0167	− 0.0146	− 0.0362	− 0.0138**	− 0.00847
*wt. = inv. dist.*	(0.00535)	(0.0140)	(0.0902)	(0.0741)	(0.00563)	(0.00787)
Wuhan	0.0153***	0.0133***	0.00826***	0.00404	0.0132***	0.0123***
*wt. = pop. flow*	(0.00273)	(0.00273)	(0.00241)	(0.00423)	(0.00222)	(0.00205)
Observations	12,096	12,096	4032	4032	8064	8064
Number of cities	288	288	288	288	288	288
Weather controls	Yes	Yes	Yes	Yes	Yes	Yes
City FE	Yes	Yes	Yes	Yes	Yes	Yes
Date FE	Yes	Yes	Yes	Yes	Yes	Yes

The dependent variable is the number of daily new cases. The endogenous explanatory variables include the average numbers of new confirmed cases in the own city and nearby cities in the preceding first and second weeks (model A) and averages in the preceding 14 days (model B). Weekly averages of daily maximum temperature, precipitation, wind speed, the interaction between precipitation and wind speed, and the inverse log distance weighted sum of these variables in other cities, during the preceding third and fourth weeks, are used as instrumental variables in the IV regressions. Weather controls include contemporaneous weather variables in the preceding first and second weeks. Standard errors in parentheses are clustered by provinces. *** *p* < 0.01, ** *p* < 0.05, * *p* < 0.1

As a robustness test, Table 5 reports the estimation results excluding the cities in Hubei province. Column (4) of Table 5 indicates that in the first sub-sample, one new case leads to 1.194 more cases within a week, while in the second sub-sample, one new case only leads to 0.899 more cases within a week. Besides, in the second subsample, one new case results in 0.250 fewer new infections between 1 and 2 weeks, which is larger in magnitude and more significant than the estimate (− 0.171) when cities in Hubei province are included for estimation (column (6) of Table 4).

**Fig. 5:**
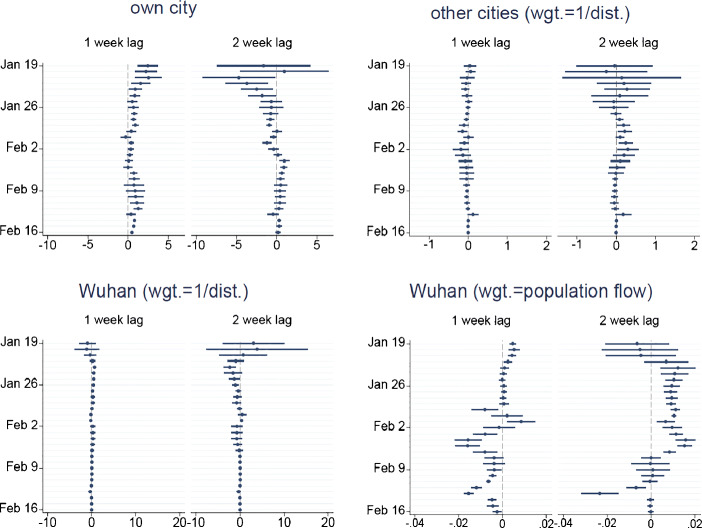
Rolling window analysis of within- and between-city transmission of COVID-19. This figure shows the estimated coefficients and 95% CIs from the instrumental variable regressions. The specification is the same as the IV regression models in Table 4. Each estimation sample contains 14 days with the starting date indicated on the horizontal axis

The time varying patterns in local transmissions are evident using the rolling window analysis (Fig. 5). The upper left panel displays the estimated coefficients on local transmissions for various 14-day sub-samples with the starting date labelled on the horizontal axis. After a slight increase in the local transmission rates, one case generally leads to fewer and fewer additional cases a few days after January 19. Besides, the transmission rate displays a slight increase beginning around February 4, which corresponds to the return travels and work resumption after Chinese Spring Festival, but eventually decreases at around February 12. Such decrease may be partly attributed to the social distancing strategies at the city level, so we examine the impacts of relevant policies in Section 5. Moreover, the transmission rates in cities outside Hubei province have been kept at low levels throughout the whole sample period (columns (4) and (6) of Table 5). These results suggest that the policies adopted at the national and provincial levels soon after January 19 prevented cities outside Hubei from becoming new hotspots of infections. Overall, the spread of the virus has been effectively contained by mid February, particularly for cities outside Hubei province.

In the epidemiology literature, the estimates on the basic reproduction number of COVID-19 are approximately within the wide range of $1.4\sim 6.5$ (Liu et al. 2020). Its value depends on the estimation method used, underlying assumptions of modeling, time period covered, geographic regions (with varying preparedness of health care systems), and factors considered in the models that affect disease transmissions (such as the behavior of the susceptible and infected population). Intuitively, it can be interpreted as measuring the expected number of new cases that are generated by one existing case. It is of interest to note that our estimates are within this range. Based on the results from model B in Tables 4 and 5, one case leads to 2.992 more cases in the same city in the next 14 days (1.876 if cities in Hubei province are excluded). In the second sub-sample (February 2–February 29), these numbers are reduced to 1.243 and 0.614, respectively, suggesting that factors such as public health measures and people’s behavior may play an important role in containing the transmission of COVID-19.

While our basic reproduction number estimate (*R*_0_) is within the range of estimates in the literature and is close to its median, five features may distinguish our estimates from some of the existing epidemiological estimates. First, our instrumental variable approach helps isolate the causal effect of virus transmissions from other confounded factors; second, our estimate is based on an extended time period of the COVID-19 pandemic (until the end of February 2020) that may mitigate potential biases in the literature that relies on a shorter sampling period within 1–28 January 2020; third, our modeling makes minimum assumptions of virus transmissions, such as imposing fewer restrictions on the relationship between the unobserved determinants of new cases and the number of cases in the past; fourth, our model simultaneously considers comprehensive factors that may affect virus transmissions, including multiple policy instruments (such as closed management of communities and shelter-at-home order), population flow, within- and between-city transmissions, economic and demographic conditions, weather patterns, and preparedness of health care system. Fifth, our study uses spatially disaggregated data that cover China (except its Hubei province), while some other studies examine Wuhan city, Hubei province, China as a whole, or overseas.

Regarding the between-city transmission from Wuhan, we observe that the population flow better explains the contagion effect than geographic proximity (Table 4). In the first sub-sample, one new case in Wuhan leads to more cases in other cities receiving more population flows from Wuhan within 1 week. Interestingly, in the second sub-sample, population flow from Wuhan significantly decreases the transmission rate within 1 week, suggesting that people have been taking more cautious measures from high COVID-19 risk areas; however, more arrivals from Wuhan in the preceding second week can still be a risk. A back of the envelope calculation indicates that one new case in Wuhan leads to 0.064 (0.050) more cases in the destination city per 10,000 travelers from Wuhan within 1 (2) week between January 19 and February 1 (February 2 and February 29)^15^. Note that while the effect is statistically significant, it should be interpreted in context. It was estimated that 15,000,000 people would travel out of Wuhan during the Lunar New Year holiday^16^. If all had gone to one city, this would have directly generated about 171 cases within 2 weeks. The risk of infection is likely very low for most travelers except for few who have previous contacts with sources of infection, and person-specific history of past contacts may be an essential predictor for infection risk, in addition to the total number of population flows^17^.

A city may also be affected by infections in nearby cities apart from spillovers from Wuhan. We find that the coefficients that represent the infectious effects from nearby cities are generally small and not statistically significant (Table 4), implying that few cities outside Wuhan are themselves exporting infections. This is consistent with the findings in the World Health Organization (2020b) that other than cases that are imported from Hubei, additional human-to-human transmissions are limited for cities outside Hubei. Restricting to cities outside Hubei province, the results are similar (Table 5), except that the transmission from Wuhan is not significant in the first half sample.

### Social and economic mediating factors

We also investigate the mediating impacts of some socioeconomic and environmental characteristics on the transmission rates (3). To ease the comparison between different moderators, we consider the mediating impacts on the influence of the average number of new cases in the past 2 weeks. Regarding own-city transmissions, we examine the mediating effects of population density, GDP per capita, number of doctors, and average temperature, wind speed, precipitation, and a dummy variable of adverse weather conditions. Regarding between-city transmissions, we consider the mediating effects of distance, difference in population density, and difference in GDP per capita since cities that are similar in density or economic development level may be more closely linked. We also include a measure of population flows from Wuhan. Table 6 reports the estimation results of the IV regressions. To ease the comparison across various moderators, for the mediating variables of within-city transmissions that are significant at 10%, we compute the changes in the variables so that the effect of new confirmed infections in the past 14 days on current new confirmed cases is reduced by 1 (columns (2) and (4)).

**Table 6:** Social and economic factors mediating the transmission of COVID-19

	(1)	(2)	(3)	(4)
	Jan 19–Feb 1		Feb 2–Feb 29	
	IV Coeff.		IV Coeff.	
Average # of new cases, previous 14 days				
Own city	− 0.251		0.672***	
	(0.977)		(0.219)	
× population density	0.000164		− 0.000202**	+ 495 per km^2^
	(0.000171)		(8.91e-05)	
× per capita GDP	0.150***	− 66, 667 RMB	0.0102	
	(0.0422)		(0.0196)	
× # of doctors	− 0.108*	+ 92, 593	0.0179	
	(0.0622)		(0.0236)	
× temperature	0.0849*	− 11.78^∘^*C*	− 0.00945	
	(0.0438)		(0.0126)	
× wind speed	− 0.109		0.128	
	(0.131)		(0.114)	
× precipitation	0.965*	− 1.04 mm	0.433*	− 2.31 mm
	(0.555)		(0.229)	
× adverse weather	0.0846		− 0.614***	+ 163*%*
	(0.801)		(0.208)	
Other cities	0.0356		− 0.00429	
*wt. = inv. distance*	(0.0375)		(0.00343)	
Other cities	0.00222		0.000192	
*wt. = inv. density ratio*	(0.00147)		(0.000891)	
Other cities	0.00232		0.00107	
*wt. = inv. per capita GDP ratio*	(0.00497)		(0.00165)	
Wuhan	− 0.165		− 0.00377	
*wt. = inv. distance*	(0.150)		(0.00981)	
Wuhan	− 0.00336		− 0.000849	
*wt. = inv. density ratio*	(0.00435)		(0.00111)	
Wuhan	− 0.440		− 0.0696	
*wt. = inv. per capita GDP ratio*	(0.318)		(0.0699)	
Wuhan	0.00729***		0.0125***	
*wt. = population flow*	(0.00202)		(0.00187)	
Observations	4032		8064	
Number of cities	288		288	
Weather controls	Yes		Yes	
City FE	Yes		Yes	
Date FE	Yes		Yes	

The dependent variable is the number of daily new confirmed cases. The sample excludes cities in Hubei province. Columns (2) and (4) report the changes in the mediating variables that are needed to reduce the impact of new confirmed cases in the preceding 2 weeks by 1, using estimates with significance levels of at least 0.1 in columns (1) and (3), respectively. The endogenous variables include the average numbers of new cases in the own city and nearby cities in the preceding 14 days and their interactions with the mediating variables. Weekly averages of daily maximum temperature, precipitation, wind speed, the interaction between precipitation and wind speed, and the inverse log distance weighted sum of these variables in neighboring cities, during the preceding third and fourth weeks, are used as instrumental variables in the IV regressions. Additional instrumental variables are constructed by interacting them with the mediating variables. Weather controls include these variables in the preceding first and second weeks. Standard errors in parentheses are clustered by provinces

*** *p* < 0.01, ** *p* < 0.05, * *p* < 0.1

In the early phase of the epidemic (January 19 to February 1), cities with more medical resources, which are measured by the number of doctors, have lower transmission rates. One standard deviation increase in the number of doctors reduces the transmission rate by 0.12. Cities with higher GDP per capita have higher transmission rates, which can be ascribed to the increased social interactions as economic activities increase^18^. In the second sub-sample, these effects become insignificant probably because public health measures and inter-city resource sharing take effects. In fact, cities with higher population density have lower transmission rates in the second sub-sample. Regarding the environmental factors, we notice different significant mediating variables across the first and second sub-samples. The transmission rates are lower with adverse weather conditions, lower temperature, or less rain. Further research is needed to identify clear mechanisms. In addition, population flow from Wuhan still poses a risk of new infections for other cities even after we account for the above mediating effects on own-city transmission. This effect is robust to the inclusion of the proximity measures based on economic similarity and geographic proximity between Wuhan and other cities. Nevertheless, we do not find much evidence on between-city transmissions among cities other than Wuhan.

## Policy response to the COVID-19 outbreak in China

**Fig. 6:**
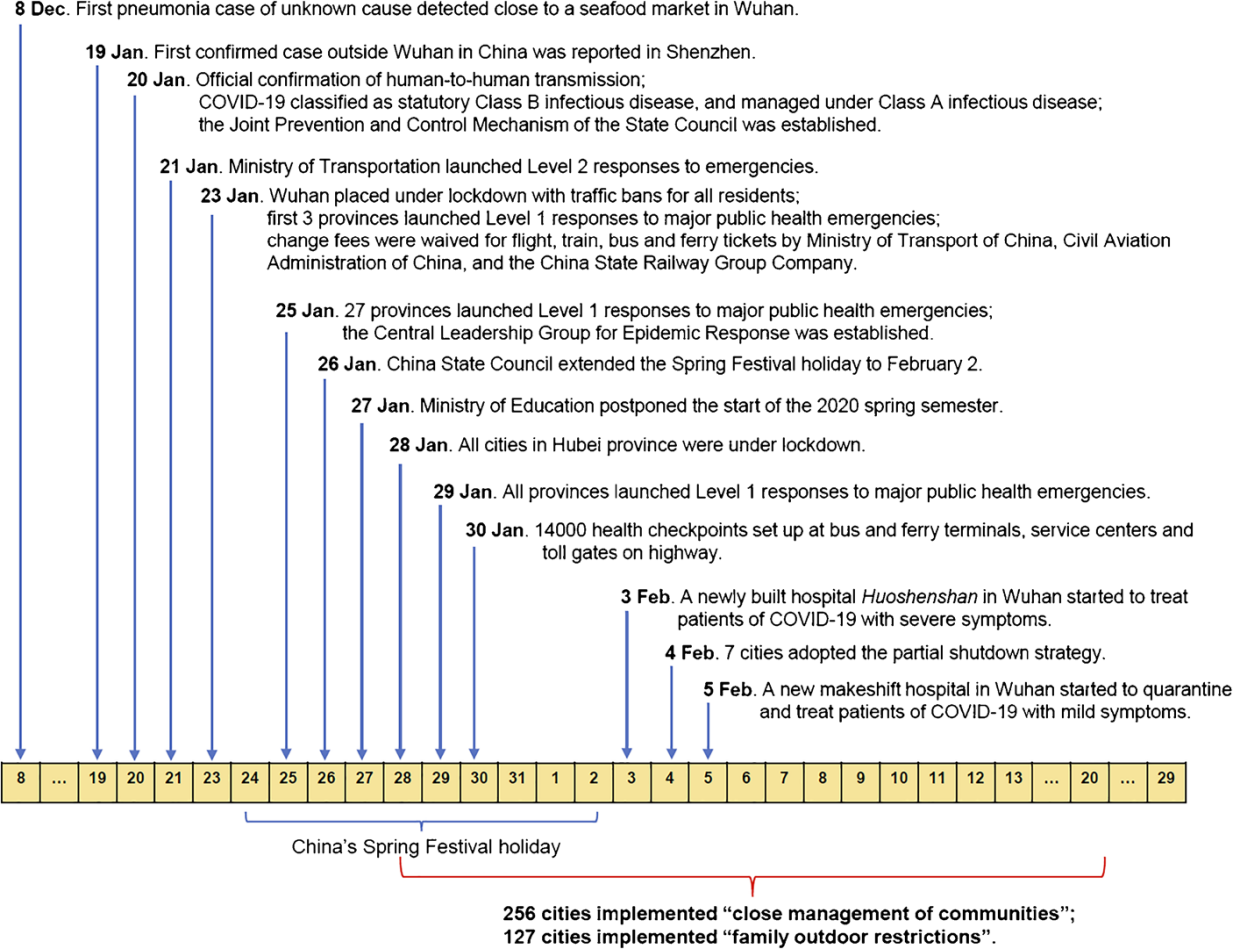
Timeline of China’s public health policies in curtailing the spread of COVID-19

As the 2002–2004 SARS outbreak has shown, non-pharmaceutical interventions (NPIs) or public health measures may decrease or effectively stop the transmission of COVID-19 even without vaccines. Although the effectiveness of a single intervention strategy can be limited, multiple interventions together may generate substantial impacts on containing the spread of the virus. Figure 6 depicts the timeline for a series of policies enacted at the national, provincial, and city levels in China since January 19. After the official confirmation of human-to-human transmission by the Chinese authorities on January 20, China has adopted a variety of NPIs to contain the COVID-19 outbreak. At the national level, COVID-19 was classified as a statutory class B infectious disease on January 20, and prevention and control measures for class A infectious diseases have been taken. Government agencies across the country were mobilized. The Joint Prevention and Control Mechanism of the State Council was established on January 20, and the Central Leadership Group for Epidemic Response was established on January 25. On January 23, National Healthcare Security Administration announced that expenses related to COVID-19 treatments would be covered by the medical insurance and the government if necessary, in order that all COVID-19 cases could be hospitalized^19^. At the provincial level, 30 provinces declared level I responses to major public health emergencies from January 23 to 25, and all provinces had declared level I responses by January 29^20^. Level I responses in China are designed for the highest state of emergencies. Measures taken include enhanced isolation and contact tracing of cases, suspension of public transport, cancelling public events, closing schools and entertainment venues, and establishment of health checkpoints (Tian et al. 2020). These policies together represent population-wide social distancing and case isolation (Ferguson et al. 2020).

### Policy response to COVID-19 in Hubei Province

Early detection of COVID-19 importation and prevention of onward transmission are crucial to all areas at risk of importation from areas with active transmissions (Gilbert et al. 2020). To contain the virus at the epicenter, Wuhan was placed under lockdown with traffic ban for all residents starting on January 23. The lockdown is not expected to be lifted until April 8. Local buses, subways, and ferries ceased operation. Ride-hailing services were prohibited, and only a limited number of taxis were allowed on road by January 24. Residents are not permitted to leave the city. Departure flights and trains were canceled at the city airport and train stations. Checkpoints were set up at highway entrances to prevent cars from leaving the city. Since January 22, it became mandatory to wear masks at work or in public places.

In addition, all cities in Hubei province implemented the lockdown policy, and most Hubei cities had also adopted measures commensurate with class A infectious diseases by January 28^21^. Residents in those areas were strongly encouraged to stay at home and not to attend any activity involving public gathering.

Health facilities in Wuhan had been extremely overstretched with shortage in medical supplies and high rates of nosocomial infections until February 2 when (1) two new hospitals, i.e., Huoshenshan and Leishenshan, were built to treat patients of COVID-19 with severe symptoms, and (2) 14 makeshift health facilities were converted to isolate patients with mild symptoms and to quarantine people suspected of contracting COVID-19, patients with fever symptoms, and close contacts of confirmed patients. This centralized treatment and isolation strategy since February 2 has substantially reduced transmission and incident cases.

However, stringent public health measures within Hubei province enforced after the massive lockdown may have little to do with virus transmissions out of Hubei province due to the complete travel ban since January 23.

### Reducing inter-city population flows

Quarantine measures have been implemented in other provinces that aim at restricting population mobility across cities and reducing the risk of importing infections^22^. Seven cities in Zhejiang, Henan, Heilongjiang, and Fujian provinces had adopted the partial shutdown strategy by February 4 (Fang et al. 2020)^23^. In Wenzhou, most public transportation was shut down, and traffic leaving the city was banned temporarily. On January 21, the Ministry of Transport of China launched level 2 responses to emergencies in order to cooperate with the National Health Commission in preventing the virus spread. On January 23, the Ministry of Transport of China, Civil Aviation Administration of China, and China State Railway Group Company, Ltd. (CSRGC) declared to waive the change fees for flight, train, bus, and ferry tickets that were bought before January 24. Later, the CSRGC extended the fee waiver policy to train tickets that were bought before February 6. By February 2, all railway stations in China had started to monitor body temperature of travelers when they enter and exit the station. Across the whole country, Transportation Departments set up 14,000 health checkpoints at bus and ferry terminals, at service centers and toll gates on highways, monitoring the body temperature of passengers and controlling the inflow of population (World Health Organization 2020b). Recent visitors to high COVID-19 risk areas are required to self-quarantine for 14 days at home or in designated facilities. On February 2, China’s Exit and Entry Administration temporarily suspended the approval and issuance of the travel permits to Hong Kong and Macau.

On January 23, Wuhan Municipal Administration of Culture and Tourism ordered all tour groups to cancel travels to Wuhan. On January 27, the Ministry of Education of China postponed start of the spring semester in 2020, and on February 7, it further announced that students were not allowed to return to school campus without approvals from school.

### Encouraging social distancing in local communities

Recent studies suggest that there is a large proportion of asymptomatic or mild-symptomatic cases, who can also spread the virus (Dong et al. 2020; Mizumoto et al. 2020; Nishiura et al. 2020; Wang et al. 2020a). Thus, maintaining social distance is of crucial importance in order to curtail the local transmission of the virus.

The period from January 24 to 31, 2020, is the traditional Chinese Spring Festival holiday, when families are supposed to get together so that inter-city travel is usually much less. People were frequently reminded by official media (via TV news and phone messages) and social media to stay at home and avoid gathering activities. On January 26, China State Council extended this holiday to February 2 to delay people’s return travel and curtail the virus spread. Nevertheless, economic activities are still supposed to resume after the spring festival, bringing people back to workplaces, which may increase the risk of virus spread.

To help local residents keep social distance and decrease the risk of virus transmissions, many cities started to implement the “closed management of communities” and “family outdoor restrictions” policies since late January (Table 7), encouraging residents to restrict nonessential travels. From January 28 to February 20, more than 250 prefecture-level cities in China implemented “closed management of communities,” which typically includes (1) keeping only one entrance for each community, (2) allowing only community residents to enter and exit the community, (3) checking body temperature for each entrant, (4) testing and quarantining cases that exhibit fever immediately, and (5) tracing and quarantining close contacts of suspicious cases. Meanwhile, residents who had symptoms of fever or dry cough were required to report to the community and were quarantined and treated in special medical facilities. Furthermore, local governments of 127 cities also imposed more stringent “family outdoor restrictions”—residents are confined or strongly encouraged to stay at home with limited exceptions, e.g., only one person in each family may go out for shopping for necessities once every 2 days^24^. Exit permits were usually distributed to each family in advance and recollected when residents reenter the community. Contacts of those patients were also traced and quarantined. Table 7 summarizes the number of cities that had imposed “closed management of communities” or “family outdoor restrictions” by different dates in February.

**Table 7:** Number of cities with local quarantine measures by different dates

Date	Closed management of communities	Family outdoor restrictions
2020-02-01	10	1
2020-02-02	20	6
2020-02-03	33	16
2020-02-04	63	38
2020-02-05	111	63
2020-02-06	155	88
2020-02-07	179	92
2020-02-08	187	98
2020-02-09	196	102
2020-02-10	215	104
2020-02-11	227	105
2020-02-12	234	108
2020-02-13	234	109
2020-02-14	235	111
2020-02-15	237	111
2020-02-16	237	122
2020-02-17	237	122
2020-02-18	238	122
2020-02-19	238	122
2020-02-20^‡^	241	123

^‡^No new cities adopt these measures after February 20

In order to help inform evidence-based COVID-19 control measures, we examine the effect of these local quarantine measures in reducing the virus transmission rates. Dummy variables for the presence of closed management of communities or family outdoor restrictions are created, and they are interacted with the number of infections in the preceding 2 weeks.

### Assessment of the effects of non-pharmaceutical interventions

Several factors may contribute to the containment of the epidemic. The transmission dynamics may change during the course of this epidemic because of improved medical treatments, more effective case isolation and contact tracing, increased public awareness, etc. Therefore, we have split the sample into two sub-samples, and the estimated coefficients can be different across the sub-samples (Section 4). NPIs such as closed management of communities, city lockdowns, and restrictions on population flow out of areas with high infection risks may also directly affect the transmission rates. While many public health measures are implemented nationwide, spatial variations exist in the adoption of two types of measures: closed management of communities (denoted by *closed management*) and family outdoor restrictions (denoted by *stay at home*), which allow us to quantify the effect of these NPIs on the transmission dynamics.

Because most of these local NPIs are adopted in February and our earlier results indicate that the transmission of COVID-19 declines during late January, we restrict the analysis sample to February 2–February 29. We also exclude cities in Hubei province, which modified the case definition related to clinically diagnosed cases on February 12 and changed the case definition related to reduced backlogs from increased capacity of molecular diagnostic tests on February 20. These modifications coincide with the adoption of local NPIs and can significantly affect the observed dynamics of confirmed cases. The adoption of *closed management* or *stay at home* is likely affected by the severity of the epidemic and correlated with the unobservables. Additional weather controls that have a good predictive power for these NPIs are selected as the instrumental variables based on the method of Belloni et al. (2016). Details are displayed in Appendix A. The estimation results of OLS and IV regressions are reported in Table 8.

**Table 8:** Effects of local non-pharmaceutical interventions

	(1)	(2)	(3)	(4)	(5)	(6)
	OLS	IV	OLS	IV	OLS	IV
Average # of new cases, 1-week lag						
Own city	0.642***	0.780***	0.684***	0.805***	0.654***	0.805***
	(0.0644)	(0.0432)	(0.0496)	(0.0324)	(0.0566)	(0.0439)
× *closed management*	− 0.593***	− 0.244***			− 0.547***	− 0.193*
	(0.162)	(0.0619)			(0.135)	(0.111)
× *stay at home*			− 0.597***	− 0.278***	− 0.0688	− 0.110
			(0.186)	(0.0800)	(0.121)	(0.143)
Other cities	0.00121	− 0.00159	0.00167	− 0.00108	0.00129	− 0.00142
*wt. = inv. dist.*	(0.000852)	(0.00167)	(0.00114)	(0.00160)	(0.000946)	(0.00183)
Wuhan	0.00184	0.00382	0.00325*	0.00443	0.00211	0.00418
*wt. = inv. dist.*	(0.00178)	(0.00302)	(0.00179)	(0.00314)	(0.00170)	(0.00305)
Wuhan	0.00298	0.00110	− 0.00187	− 0.000887	0.00224	− 3.26e-07
*wt. = pop. flow*	(0.00264)	(0.00252)	(0.00304)	(0.00239)	(0.00254)	(0.00260)
Average # of new cases, 2-week lag						
Own city	0.0345	− 0.0701	− 0.0103	− 0.0818	0.0396	− 0.0533
	(0.0841)	(0.0550)	(0.0921)	(0.0523)	(0.0804)	(0.0678)
× *closed management*	− 0.367***	− 0.103			− 0.259**	0.0344
	(0.0941)	(0.136)			(0.111)	(0.222)
× *stay at home*			− 0.294***	− 0.102	− 0.124*	− 0.162
			(0.0839)	(0.136)	(0.0720)	(0.212)
Other cities	− 0.00224	− 0.00412**	− 0.00190	− 0.00381**	− 0.00218	− 0.00397**
*wt. = inv. dist.*	(0.00135)	(0.00195)	(0.00118)	(0.00177)	(0.00129)	(0.00192)
Wuhan	− 0.00512	0.00197	− 0.00445	0.00231	− 0.00483	0.00227
*wt. = inv. dist.*	(0.00353)	(0.00367)	(0.00328)	(0.00348)	(0.00340)	(0.00376)
Wuhan	0.00585***	0.00554***	0.00534***	0.00523***	0.00564***	0.00516***
*wt. = pop. flow*	(0.00110)	(0.000929)	(0.00112)	(0.00104)	(0.00109)	(0.00116)
Observations	8064	8064	8064	8064	8064	8064
Number of cities	288	288	288	288	288	288
Weather controls	Yes	Yes	Yes	Yes	Yes	Yes
City FE	Yes	Yes	Yes	Yes	Yes	Yes
Date FE	Yes	Yes	Yes	Yes	Yes	Yes

The sample is from February 2 to February 29, excluding cities in Hubei province. The dependent variable is the number of daily new confirmed cases. The instrumental variables include weekly averages of daily maximum temperature, wind speed, precipitation, and the interaction between wind speed and precipitation, in the preceding third and fourth weeks, and the inverse log distance weighted averages of these variables in other cities. Additional instrumental variables are constructed by interacting these excluded instruments with variables that predict the adoption of closed management of communities or family outdoor restrictions (Table 10). The weather controls include weather characteristics in the preceding first and second weeks. Standard errors in parentheses are clustered by provinces. *** *p* < 0.01, ** *p* < 0.05, * *p* < 0.1

We find that *closed management* and *stay at home* significantly decrease the transmission rates. As a result of closed management of communities, one infection will generate 0.244 (95% CI, $-0.366\sim -0.123$) fewer new infections in the first week. The effect in the second week is also negative though not statistically significant. Family outdoor restrictions (*stay at home*) are more restrictive than closing communities to visitors and reduce additional infections from one infection by 0.278 (95% CI, $-0.435\sim -0.121$) in the first week. The effect in the second week is not statistically significant. To interpret the magnitude of the effect, it is noted that the reproduction number of SARS-CoV-2 is estimated to be around $1.4\sim 6.5$ as of January 28, 2020 (Liu et al. 2020).

Many cities implement both policies. However, it is not conclusive to ascertain the effect of further imposing family outdoor restrictions in cities that have adopted closed management of communities. When both policies are included in the model, the OLS coefficients (column (5)) indicate that *closed management* reduces the transmission rate by 0.547 (95% CI, $-0.824\sim -0.270$) in the first week, and by 0.259 (95% CI, $-0.485\sim -0.032$) in the second week, while the additional benefit from *stay at home* is marginally significant in the second week (− 0.124, 95% CI, $-0.272\sim 0.023$). The IV estimates indicate that *closed management* reduces the transmission rate in the first week by 0.193 (95% CI, $-0.411\sim 0.025$), while the effect in the second week and the effects of *stay at home* are not statistically significant. Additional research that examines the decision process of health authorities or documents the local differences in the actual implementation of the policies may offer insights into the relative merits of the policies.

We further assess the effects of NPIs by conducting a series of counterfactual exercises. After estimating (3) by 2SLS, we obtain the residuals. Then, the changes in *y*_*ct*_ are predicted for counterfactual changes in the transmission dynamics (i.e., coefficients $\alpha _{\text {within},\tau }^{k}$) and the impositions of NPIs (i.e., $\bar {h}_{ct}^{k\tau }$, and the lockdown of Wuhan $\bar {m}_{c,\text {Wuhan},t}^{k\tau }$). In scenario A, no cities adopted family outdoor restrictions (*stay at home*). Similarly, in scenario B, no cities implemented closed management of communities. We use the estimates in columns (2) and (4) of Table 8 to conduct the counterfactual analyses for scenarios A and B, respectively. In scenario C, we assume that the index of population flows out of Wuhan after the Wuhan lockdown (January 23) took the value that was observed in 2019 for the same lunar calendar date (Fig. 3), which would be plausible had there been no lockdown around Wuhan. It is also likely that in the absence of lockdown but with the epidemic, more people would leave Wuhan compared with last year (Fang et al. 2020), and the effect would then be larger. In scenario D, we assume that the within-city transmission dynamics were the same as those observed between January 19 and February 1, i.e., the coefficient of 1-week lag own-city infections was 2.456 and the coefficient of 2-week lag own-city infections was − 1.633 (column (4) of Table 4), which may happen if the transmission rates in cities outside Hubei increased in the same way as those observed for cities in Hubei. Appendix C contains the technical details on the computation of counterfactuals.

**Fig. 7:**
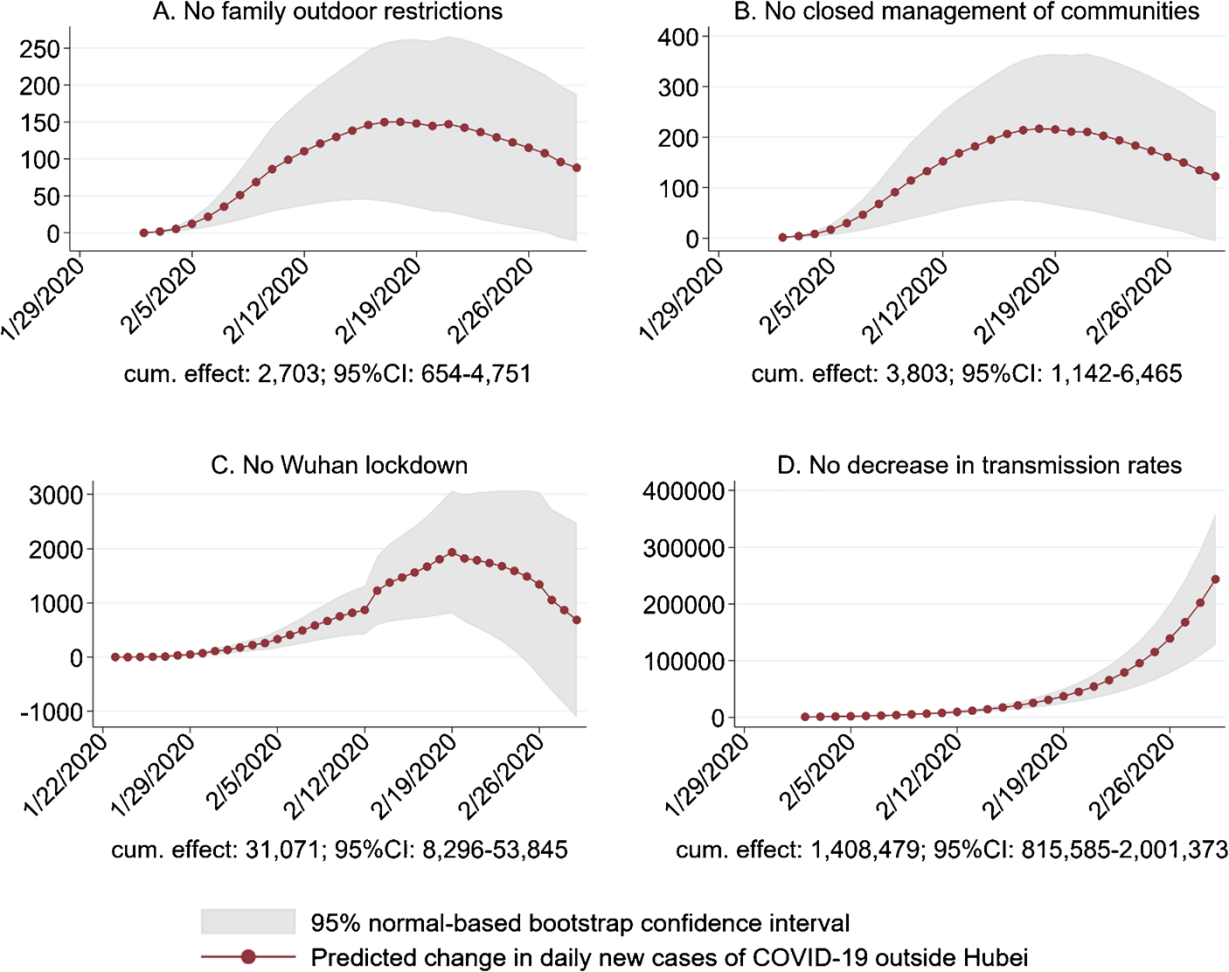
Counterfactual policy simulations. This figure displays the daily differences between the total predicted number and the actual number of daily new COVID-19 cases for each of the four counterfactual scenarios for cities outside Hubei province in mainland China. The spike on February 12 in scenario C is due to a sharp increase in daily case counts in Wuhan resulting from changes in case definitions in Hubei province (see Appendix B for details)

In Fig. 7, we report the differences between the predicted number of daily new cases in the counterfactual scenarios and the actual data, for cities outside Hubei province. We also report the predicted cumulative effect in each scenario at the bottom of the corresponding panel in Fig. 7. Had the transmission rates in cities outside Hubei province increased to the level observed in late January, by February 29, there would be 1,408,479 (95% CI, $815,585\sim 2,001,373$) more cases (scenario D). Assuming a fatality rate of 4%, there would be 56,339 more deaths. The magnitude of the effect from Wuhan lockdown and local NPIs is considerably smaller. As a result of Wuhan lockdown, 31,071 (95% CI, $8296\sim 53,845$) fewer cases would be reported for cities outside Hubei by February 29 (scenario C). Closed management of communities and family outdoor restrictions would reduce the number of cases by 3803 (95% CI, $1142\sim 6465$; or 15.78 per city with the policy) and 2703 (95% CI, $654\sim 4751$; or 21.98 per city with the policy), respectively. These estimates, combined with additional assumptions on the value of statistical life, lost time from work, etc., may contribute to cost-benefit analyses of relevant public health measures.

Our counterfactual simulations indicate that suppressing local virus transmissions so that transmission rates are kept well below those observed in Hubei in late January is crucial in forestalling large numbers of infections for cities outside Hubei. Our retrospective analysis of the data from China complements the simulation study of Ferguson et al. (2020). Our estimates indicate that suppressing local transmission rates at low levels might have avoided one million or more infections in China. Chinazzi et al. (2020) also find that reducing local transmission rates is necessary for effective containment of COVID-19. The public health policies announced by the national and provincial authorities in the last 2 weeks in January may have played a determinant role (Tian et al. 2020) in keeping local transmission rates in cities outside Hubei at low levels throughout January and February. Among the measures implemented following provincial level I responses, Shen et al. (2020) highlight the importance of contact tracing and isolation of close contacts before onset of symptoms in preventing a resurgence of infections once the COVID-19 suppression measures are relaxed. We also find that travel restrictions on high-risk areas (the lockdown in Wuhan), and to a lesser extent, closed management of communities and family outdoor restrictions, further reduce the number of cases. It should be noted that these factors may overlap in the real world. In the absence of the lockdown in Wuhan, the health care systems in cities outside Hubei could face much more pressure, and local transmissions may have been much higher. In China, the arrival of the COVID-19 epidemic coincided with the Lunar New Year for many cities. Had the outbreak started at a different time, the effects and costs of these policies would likely be different.

## Conclusion

This paper examines the transmission dynamics of the coronavirus disease 2019 in China, considering both within- and between-city transmissions. Our sample is from January 19 to February 29 and covers key episodes such as the initial spread of the virus across China, the peak of infections in terms of domestic case counts, and the gradual containment of the virus in China. Changes in weather conditions induce exogenous variations in past infection rates, which allow us to identify the causal impact of past infections on new cases. The estimates suggest that the infectious effect of the existing cases is mostly observed within 1 week and people’s responses can break the chain of infections. Comparing estimates in two sub-samples, we observe that the spread of COVID-19 has been effectively contained by mid February, especially for cities outside Hubei province. Data on real-time population flows between cities have become available in recent years. We show that this new source of data is valuable in explaining between-city transmissions of COVID-19, even after controlling for traditional measures of geographic and economic proximity.

By April 5 of 2020, COVID-19 infections have been reported in more than 200 countries or territories and more than 64,700 people have died. Behind the grim statistics, more and more national and local governments are implementing countermeasures. Cross border travel restrictions are imposed in order to reduce the risk of case importation. In areas with risks of community transmissions, public health measures such as social distancing, mandatory quarantine, and city lockdown are implemented. In a series of counterfactual simulations, we find that based on the experience in China, preventing sustained community transmissions from taking hold in the first place has the largest impact, followed by restricting population flows from areas with high risks of infections. Local public health measures such as closed management of communities and family outdoor restrictions can further reduce the number of infections.

A key limitation of the paper is that we are not able to disentangle the effects from each of the stringent measures taken, as within this 6-week sampling period, China enforced such a large number of densely timed policies to contain the virus spreading, often simultaneously in many cities. A second limitation is that shortly after the starting date of the official data release for confirmed infected cases throughout China, i.e., January 19, 2020, many stringent measures were implemented, which prevents researchers to compare the post treatment sub-sample with a pre treatment sub-sample during which no strict policies were enforced. Key knowledge gaps remain in the understanding of the epidemiological characteristics of COVID-19, such as individual risk factors for contracting the virus and infections from asymptotic cases. Data on the demographics and exposure history for those who have shown symptoms as well as those who have not will help facilitate these research.

## Figures and Tables

**Fig. 8:**
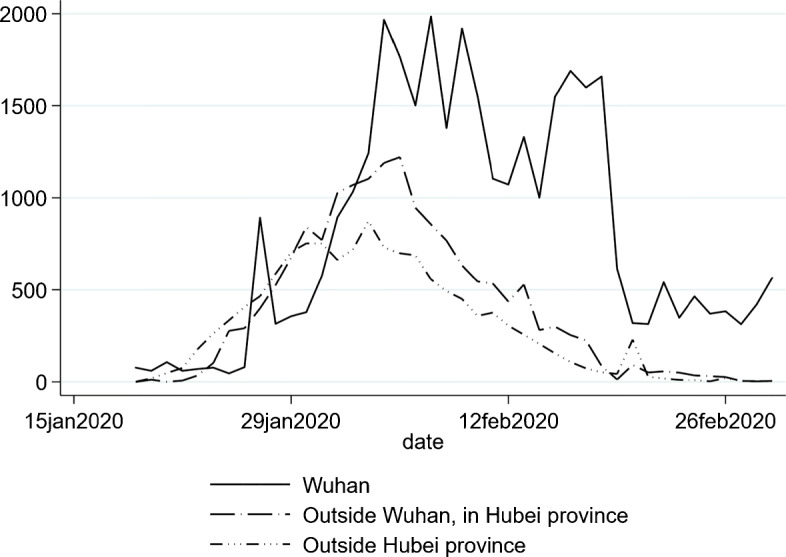
Number of daily new confirmed cases of COVID-19 in mainland China and revised case counts in Hubei Province

## Appendix

The Appendix consists of three sections. Section A provides details on the first stage of the IV regressions and the selection of the instrumental variables for the local public health policies. Section B shows that our main findings are not sensitive to the adjustment in COVID-19 case definitions in Hubei province in February. Section A contains details on the computation of the counterfactuals.

## Appendix A. First stage regressions

Weather conditions affect disease transmissions either directly if the virus can more easily survive and spread in certain environment, or indirectly by changing human behavior. Table 9 reports results of the first stage of the IV regressions (Table 4) using the full sample. In columns (1) and (2), the dependent variables are the numbers of newly confirmed COVID-19 cases in the own city in the preceding first and second weeks, respectively. In columns (3) and (4), the dependent variables are the sum of inverse log distance weighted numbers of newly confirmed COVID-19 cases in other cities in the preceding first and second weeks, respectively. These are the endogenous variables in the IV regressions. The weather variables in the preceding first and second weeks are included in the control variables. The weather variables in the preceding third and fourth weeks are the excluded instruments, and their coefficients are reported in the table. Because the variables are averages in 7-day moving windows, the error term may be serially correlated, and we include city by week fixed effects. Also included in the control variables are the average numbers of new cases in Wuhan in the preceding first and second weeks, interacted with the inverse log distance or the population flow.

**Table 9:** First stage regressions

Dependent variable	Average # of new cases			
	Own city		Other cities	
	1-week lag	2-week lag	1-week lag	2-week lag
	(1)	(2)	(3)	(4)
*Own City*				
Maximum temperature, 3-week lag	0.200***	− 0.0431	0.564	− 2.022***
	(0.0579)	(0.0503)	(0.424)	(0.417)
Precipitation, 3-week lag	− 0.685	− 0.865*	4.516	− 1.998
	(0.552)	(0.480)	(4.045)	(3.982)
Wind speed, 3-week lag	0.508**	0.299	− 0.827	3.247*
	(0.256)	(0.223)	(1.878)	(1.849)
Precipitation × wind speed, 3-week lag	− 0.412**	0.122	− 1.129	− 2.091
	(0.199)	(0.173)	(1.460)	(1.437)
Maximum temperature, 4-week lag	0.162***	0.125**	1.379***	1.181***
	(0.0560)	(0.0487)	(0.410)	(0.404)
Precipitation, 4-week lag	0.0250	− 0.503	2.667	8.952***
	(0.440)	(0.383)	(3.224)	(3.174)
Wind speed, 4-week lag	0.179	0.214	− 1.839	1.658
	(0.199)	(0.173)	(1.458)	(1.435)
Precipitation × wind speed, 4-week lag	− 0.354**	− 0.0270	1.107	− 2.159**
	(0.145)	(0.126)	(1.059)	(1.043)
*Other cities, weight = inverse distance*				
Maximum temperature, 3-week lag	− 0.0809***	− 0.00633	0.0520	1.152***
	(0.0203)	(0.0176)	(0.149)	(0.146)
Precipitation, 3-week lag	4.366***	− 2.370***	17.99***	− 72.68***
	(0.639)	(0.556)	(4.684)	(4.611)
Wind speed, 3-week lag	0.326***	− 0.222**	− 1.456	− 11.02***
	(0.126)	(0.110)	(0.926)	(0.912)
Precipitation × wind speed, 3-week lag	− 1.780***	0.724***	− 6.750***	27.73***
	(0.227)	(0.197)	(1.663)	(1.637)
Maximum temperature, 4-week lag	− 0.0929***	− 0.0346*	− 0.518***	0.0407
	(0.0220)	(0.0191)	(0.161)	(0.159)
Precipitation, 4-week lag	3.357***	− 0.578	46.57***	− 25.31***
	(0.504)	(0.438)	(3.691)	(3.633)
Wind speed, 4-week lag	0.499***	0.214**	4.660***	− 4.639***
	(0.107)	(0.0934)	(0.787)	(0.774)
Precipitation × wind speed, 4-week lag	− 1.358***	− 0.0416	− 17.26***	8.967***
	(0.178)	(0.155)	(1.303)	(1.282)
*F* statistic	11.41	8.46	19.10	36.32
*p* value	0.0000	0.0000	0.0000	0.0000
Observations	12,768	12,768	12,768	12,768
Number of cities	304	304	304	304
# cases in Wuhan	Yes	Yes	Yes	Yes
Contemporaneous weather controls	Yes	Yes	Yes	Yes
City FE	Yes	Yes	Yes	Yes
Date FE	Yes	Yes	Yes	Yes
City by week FE	Yes	Yes	Yes	Yes

This table shows the results of the first stage IV regressions. The weather variables are weekly averages of daily weather readings. Coefficients of the weather variables which are used as excluded instrumental variables are reported. *** *p* < 0.01, ** *p* < 0.05, * *p* < 0.1

Because the spread of the virus depends on both the number of infectious people and the weather conditions, the coefficients in the first stage regressions do not have structural interpretations. The Wald tests on the joint significance of the excluded instruments are conducted and their *F* statistics are reported. The excluded instruments have good predictive power.

The implementation of local public health measures is likely correlated with the extent of the virus spread, so weather conditions that affect virus transmissions may also affect the likelihood that the policy is adopted. The influence of weather conditions on policy adoption may be complicated, so we use the Cluster-Lasso method of Belloni et al. (2016) to select the weather variables that have good predictive power on the adoption of closed management of communities or family outdoor restrictions. Let *d*_*c**t*_ be the dummy variable of the adoption of the local public health measure, i.e., *d*_*c**t*_ = 1 if the policy is in place in city *c* at day *t*. *q*_*c**t*_ is a vector of candidate weather variables, including weekly averages of daily mean temperature, maximum temperature, minimum temperature, dew point, station-level pressure, sea-level pressure, visibility, wind speed, maximum wind speed, snow depth, precipitation, dummy for adverse weather conditions, squared terms of these variables, and interactions among them. First, city and day fixed effects are removed. $\ddot {d}_{ct}=d_{ct}-\frac {1}{n}{\sum }_{c}d_{ct}-\frac {1}{T}{\sum }_{t}d_{ct}+\frac {1}{nT}{\sum }_{ct}d_{ct}$ and $\ddot {q}_{ct}$ is defined similarly. The Cluster-Lasso method solves the following minimization problem:

$$ \frac{1}{nT}\sum\limits_{ct}\left( \ddot{d}_{ct}-\ddot{q}_{ct}^{\prime}b\right)^{2} + \frac{\lambda}{nT}\sum\limits_{k}\phi_{k}|b_{k}|. $$

*λ* and *ϕ* are penalty parameters. A larger penalty value forces more coefficients to zero. The penalty parameters are picked using the theoretical result of Belloni et al. (2016). The estimation uses the Stata package by Ahrens et al. (2019). Table 10 lists the selected weather variables, which are used as the instruments in Table 8.

**Table 10:** Variables selected

Dependent variable: closed management of communities	
Dew point	1-week lag
Diurnal temperature range	1-week lag
Dew point	2-week lag
Sea-level pressure	2-week lag
Dew point	3-week lag
Visibility	4-week lag
Precipitation	4-week lag
Dependent variable: family outdoor restrictions	
Station pressure	1-week lag
Dummy for adverse weather conditions such as fog, rain, and drizzle	1-week lag
Maximum temperature	2-week lag
Sea-level pressure	2-week lag
Average temperature	3-week lag
Minimum temperature	3-week lag
Visibility	3-week lag

This table shows the weather variables selected by *lassopack* (Ahrens et al. 2019), which implements the Cluster-Lasso method of Belloni et al. (2016). City and date fixed effects are included. Candidate variables include weekly averages of daily mean temperature, maximum temperature, minimum temperature, dew point, station-level pressure, sea-level pressure, visibility, wind speed, maximum wind speed, snow depth, precipitation, dummy for adverse weather conditions, squared terms of these variables, and interactions among them

## Appendix B. Exclude clinically diagnosed cases in Hubei

COVID-19 case definitions were changed in Hubei province on February 12 and February 20. Starting on February 12, COVID-19 cases could also be confirmed based on clinical diagnosis in Hubei province, in addition to molecular diagnostic tests. This resulted in a sharp increase in the number of daily new cases reported in Hubei, and in particular Wuhan (Fig. 2). The use of clinical diagnosis in confirming cases ended on February 20. The numbers of cases that are confirmed based on clinical diagnosis for February 12, 13, and 14 are reported by the Health Commission of Hubei Province and are displayed in Table 11. As a robustness check, we re-estimate the model after removing these cases from the daily case counts (Fig. 8). Our main findings still hold (Table 12). The transmission rates are significantly lower in February compared with January. Population flow from the epidemic source increases the infections in destinations, and this effect is slightly delayed in February.

**Table 11:** Number of cumulative clinically diagnosed cases in Hubei

City	Feb 12	Feb 13	Feb 14
Ezhou	155	168	189
Enshi	19	21	27
Huanggang	221	306	306
Huangshi	12	26	42
Jingmen	202	155^‡^	150^‡^
Jingzhou	287	269^‡^	257^‡^
Qianjiang	0	9	19
Shiyan	3	4	3^‡^
Suizhou	0	6	4^‡^
Tianmen	26	67	65^‡^
Wuhan	12364	14031	14953
Xiantao	2	2	2
Xianning	6	189	286
Xiangyang	0	0	4
Xiaogan	35	80	148
Yichang	0	51	67

^‡^The reductions in cumulative case counts are due to revised diagnosis from further tests

**Table 12:** Within- and between-city transmission of COVID-19, revised case counts in Hubei Province

	Jan 19–Feb 29		Jan 19–Feb 1		Feb 2–Feb 29	
	(1)	(2)	(3)	(4)	(5)	(6)
	OLS	IV	OLS	IV	OLS	IV
Model A: lagged variables are averages over the preceding first and second week separately						
Average # of new cases, 1-week lag						
Own city	0.747***	0.840***	0.939***	2.456***	0.790***	1.199***
	(0.0182)	(0.0431)	(0.102)	(0.638)	(0.0211)	(0.0904)
Other cities	0.00631**	0.0124	0.0889	0.0412	− 0.00333	− 0.0328
*wt. = inv. dist.*	(0.00289)	(0.00897)	(0.0714)	(0.0787)	(0.00601)	(0.0230)
Wuhan	0.0331***	0.0277	− 0.879	− 0.957	0.0543*	0.0840
*wt. = inv. dist.*	(0.0116)	(0.0284)	(0.745)	(0.955)	(0.0271)	(0.0684)
Wuhan	0.00365***	0.00408***	0.00462***	0.00471***	− 0.000882	− 0.00880***
*wt. = pop. flow*	(0.000282)	(0.000287)	(0.000326)	(0.000696)	(0.000797)	(0.00252)
Average # of new cases, 2-week lag						
Own city	− 0.519***	− 0.673***	2.558	− 1.633	− 0.286***	− 0.141
	(0.0138)	(0.0532)	(2.350)	(2.951)	(0.0361)	(0.0899)
Other cities	− 0.00466	− 0.0208	− 0.361	− 0.0404	− 0.00291	− 0.0235**
*wt. = inv. dist.*	(0.00350)	(0.0143)	(0.371)	(0.496)	(0.00566)	(0.0113)
Wuhan	− 0.0914*	0.0308	3.053	3.031	− 0.154	0.0110
*wt. = inv. dist.*	(0.0465)	(0.0438)	(2.834)	(3.559)	(0.0965)	(0.0244)
Wuhan	0.00827***	0.00807***	0.00711***	− 0.00632	0.0119***	0.0112***
*wt. = pop. flow*	(0.000264)	(0.000185)	(0.00213)	(0.00741)	(0.000523)	(0.000627)
Model B: lagged variables are averages over the preceding 2 weeks						
Own city	0.235***	0.983***	1.564***	2.992***	0.391***	0.725***
	(0.0355)	(0.158)	(0.174)	(0.892)	(0.0114)	(0.101)
Other cities	0.00812	− 0.0925*	0.0414	0.0704	0.0181	− 0.00494
*wt. = inv. dist.*	(0.00899)	(0.0480)	(0.0305)	(0.0523)	(0.0172)	(0.0228)
Wuhan	− 0.172*	− 0.114**	− 0.309	− 0.608	− 0.262	− 0.299*
*wt. = inv. dist.*	(0.101)	(0.0472)	(0.251)	(0.460)	(0.161)	(0.169)
Wuhan	0.0133***	0.0107***	0.00779***	0.00316	0.0152***	0.0143***
*wt. = pop. flow*	(0.000226)	(0.000509)	(0.000518)	(0.00276)	(0.000155)	(0.000447)
Observations	12,768	12,768	4,256	4,256	8,512	8,512
Number of cities	304	304	304	304	304	304
Weather controls	Yes	Yes	Yes	Yes	Yes	Yes
City FE	Yes	Yes	Yes	Yes	Yes	Yes
Date FE	Yes	Yes	Yes	Yes	Yes	Yes

The dependent variable is the number of daily new cases. The endogenous explanatory variables include the average numbers of new confirmed cases in the own city and nearby cities in the preceding first and second weeks (model A) and averages in the preceding 14 days (model B). Weekly averages of daily maximum temperature, precipitation, wind speed, the interaction between precipitation and wind speed, and the inverse log distance weighted sum of these variables in other cities, during the preceding third and fourth weeks, are used as instrumental variables in the IV regressions. Weather controls include contemporaneous weather variables in the preceding first and second weeks. Standard errors in parentheses are clustered by provinces. *** *p* < 0.01, ** *p* < 0.05, * *p* < 0.1

## Appendix C. Computation of counterfactuals

Our main model is

4 $$ \begin{array}{@{}rcl@{}} y_{ct}&= & \sum\limits_{\tau=1}^{2}\sum\limits_{k=1}^{K_{\text{within}}}\alpha_{\text{within},\tau}^{k}\bar{h}_{ct}^{k\tau}\bar{y}_{ct}^{\tau}+\sum\limits_{\tau=1}^{2}\sum\limits_{k=1}^{K_{\text{between}}}{\sum}_{r\neq c}\alpha_{\text{between},\tau}^{k}\bar{m}_{crt}^{k\tau}\bar{y}_{rt}^{\tau}+\sum\limits_{\tau=1}^{2}\sum\limits_{k=1}^{K_{\text{Wuhan}}}\rho_{\tau}^{k}\bar{m}_{c,\text{Wuhan},t}^{k\tau}\bar{z}_{t}^{\tau}\\ & + &x_{ct}\upbeta+\epsilon_{ct}. \end{array} $$

It is convenient to write it in vector form,

5 $$ Y_{nt} = \sum\limits_{s=1}^{14}\left( H_{nt,s}(\alpha_{\text{within}})+M_{nt,s}(\alpha_{\text{between}})\right)Y_{n,t-s} + \sum\limits_{\tau=1}^{2}Z_{nt}^{\tau}\rho_{\tau} + X_{nt}\upbeta + \epsilon_{nt}, $$

where $Y_{nt} = \left (\begin {array}{lll}y_{1t} & {\cdots } & y_{nt} \end {array}\right )^{\prime }$ and *𝜖*_*n**t*_ are *n* × 1 vectors. Assuming that *Y*_*n**s*_ = 0 if *s* ≤ 0, because our sample starts on January 19, and no laboratory confirmed case was reported before January 19 in cities outside Wuhan. $X_{nt}=\left (\begin {array}{lll}x_{1t}^{\prime } & {\cdots } x_{nt}^{\prime } \end {array}\right )^{\prime }$ is an *n* × *k* matrix of the control variables. *H*_*n**t*,*s*_(*α*_within_) is an *n* × *n* diagonal matrix corresponding to the *s*-day time lag, with parameters $\alpha _{\text {within}} = \{\alpha _{\text {within},\tau }^{k}\}_{k=1,\cdots ,K_{\text {within}},\tau = 1,2}$. For example, for *s* = 1,⋯ , 7, the *i* th diagonal element of *H*_*n**t*,*s*_(*α*_within_) is $\frac {1}{7}{\sum }_{k=1}^{K_{\text {within}}}\alpha _{\text {within},1}^{k}\bar {h}_{ct,i}^{k1}$, and for *s* = 8,⋯ , 14, the *i* th diagonal element of *H*_*n**t*,*s*_(*α*_within_) is $\frac {1}{7}{\sum }_{k=1}^{K_{\text {within}}}\alpha _{\text {within},2}^{k}\bar {h}_{ct,i}^{k2}$. *M*_*n**t*,*s*_(*α*_between_) is constructed similarly. For example, for *s* = 1,⋯ , 7 and *i*≠*j*, the *ij* th element of *M*_*n**t*,*s*_(*α*_between_) is $\frac {1}{7}{\sum }_{k=1}^{K_{\text {between}}}\alpha _{\text {between},1}^{k}\bar {m}_{ijt}^{k1}$. $Z_{nt}^{\tau }$ is an *n* × *K*_Wuhan_ matrix corresponding to the transmission from Wuhan. For example, the *ik* th element of $Z_{nt}^{1}$ is $\bar {m}_{i,\text {Wuhan},t}^{k1}\bar {z}_{t}^{1}$.

We first estimate the parameters in Eq. 4 by 2SLS and obtain the residuals $\hat {\epsilon }_{n1},\cdots ,\hat {\epsilon }_{nT}$. Let $\hat {\cdot }$ denote the estimated value of parameters and $\tilde {\cdot }$ denote the counterfactual changes. The counterfactual value of *Y*_*n**t*_ is computed recursively,

$$ \begin{array}{@{}rcl@{}} \tilde{Y}_{n1} & =& \sum\limits_{\tau=1}^{2}\tilde{Z}_{n1}^{\tau}\hat{\rho}_{\tau} + X_{n1}\hat{\upbeta} + \hat{\epsilon}_{n1}, \\ \tilde{Y}_{n2} & =& \sum\limits_{s=1}^{1}\left( \tilde{H}_{n2,s}(\hat{\alpha}_{\text{within}})+\tilde{M}_{n2,s}(\hat{\alpha}_{\text{between}})\right)\tilde{Y}_{n,2-s} + \sum\limits_{\tau=1}^{2}\tilde{Z}_{n2}^{\tau}\hat{\rho}_{\tau} + X_{n2}\hat{\upbeta} + \hat{\epsilon}_{n2}, \\ \tilde{Y}_{n3} & =& \sum\limits_{s=1}^{2}\left( \tilde{H}_{n3,s}(\hat{\alpha}_{\text{within}})+\tilde{M}_{n3,s}(\hat{\alpha}_{\text{between}})\right)\tilde{Y}_{n,3-s} + \sum\limits_{\tau=1}^{2}\tilde{Z}_{n3}^{\tau}\hat{\rho}_{\tau} + X_{n3}\hat{\upbeta} + \hat{\epsilon}_{n3}, \\ &{\vdots}   & \end{array} $$

The counterfactual change for date *t* is ${\Delta } Y_{nt} = \tilde {Y}_{nt} - Y_{nt}$. The standard error of Δ*Y*_*n**t*_ is obtained from 1000 bootstrap iterations. In each bootstrap iteration, cities are sampled with replacement and the model is estimated to obtain the parameters. The counterfactual predictions are obtained using the above equations with the estimated parameters and the counterfactual scenario (e.g., no cities adopted lockdown).

## Footnotes

1 COVID-19 is also known as novel coronavirus pneumonia or 2019-nCoV acute respiratory disease.

2 In 2020, the Lunar New Year was on January 25.

3 Coronavirus COVID-19 Global Cases by the Center for Systems Science and Engineering (CSSE) at Johns Hopkins University (https://gisanddata.maps.arcgis.com/apps/opsdashboard/index.html#/bda7594740fd40299423467b48e9ecf6).

4 For instance, using data on the first 425 COVID-19 patients by January 22, Li et al. (2020) estimate a basic reproduction number of 2.2. Based on time-series data on the number of COVID-19 cases in mainland China from January 10 to January 24, Zhao et al. (2020) estimate that the mean reproduction number ranges from 2.24 to 3.58.

5 We assume a case fatality rate of 4%, the same as China’s current average level. Of course, the eventual case fatality rate may be different from the current value, and it depends on many key factors, such as the preparedness of health care systems and the demographic structure of the population outside Hubei province in comparison to China as a whole. Also importantly, among patients who have died from COVID-19, the time from symptom onset to outcome ranges from 2 to 8 weeks (World Health Organization 2020b), which is partially beyond the time window of this analysis. Therefore, we defer more rigorous estimates about avoided fatality to future studies.

6 Li et al. (2020) document the exposure history of the first 425 cases. It is suspected that the initial cases were linked to the Huanan Seafood Wholesale Market in Wuhan.

7 The COVID-19 epidemic is still ongoing at the time of writing, and the estimates are revised from time to time in the literature as new data become available. The current estimates include the following. The incubation period is estimated to be between 2 and 10 days (World Health Organization 2020a), 5.2 days (Li et al. 2020), or 3 days (median, Guan et al. (2020)). The average infectious period is estimated to be 1.4 days (Wu et al. 2020a).

8 On February 12, cities in Hubei province include clinically diagnosed cases in the confirmed cases, in addition to cases that are confirmed by nucleic acid tests, which results in a sharp increase in the number of confirmed cases for cities in Hubei on February 12. The common effect on other cities is controlled for by the day fixed effect.

9 Flu viruses are easier to survive in cold weather. Adverse weather conditions also limit outdoor activities which can decrease the chance of contracting the virus. For details, see Adda (2016) and Section 3.2.

10 Hong Kong and Macao are excluded from our analysis due to the lack of some socioeconomic variables.

11 Baidu migration (https://qianxi.baidu.com).

12 The 100-km circle is consistent with the existing literature. Most studies on the socioeconomic impacts of climate change have found that estimation results are insensitive to the choice of the cutoff distance (Zhang et al. 2017).

13 These cities are Xiaogan, Huanggang, Jingzhou, Suizhou, Ezhou, and Xiangyang.

14 The shares of top 100 destinations are available. The starting and ending dates of the average shares released by Baidu do not precisely match the period of the analysis sample.

15 It is estimated that 14,925,000 people traveled out of Wuhan in 2019 during the Lunar New Year holiday (http://www.whtv.com.cn/p/17571.html). The sum of Baidu’s migration index for population flow out of Wuhan during the 40 days around the 2019 Lunar New Year is 203.3, which means one index unit represents 0.000013621 travelers. The destination share is in percentage. With one more case in Wuhan, the effect on a city receiving 10,000 travelers from Wuhan is 0.00471 × 0.000013621 × 100 × 10000 = 0.064.

16 http://www.whtv.com.cn/p/17571.html

17 From mid February, individual specific health codes such as Alipay Health Code and WeChat Health Code are being used in many cities to aid quarantine efforts.

18 Disease prevalence can also affect economic development. One channel is the fertility decision which leads to changes in the demographic structure (e.g., Durevall and Lindskog 2011; Chin and Wilson 2018). Fogli and Veldkamp (forthcoming) show that because a dense network spreads diseases faster and higher income is positively correlated with more closely connected social network, infectious diseases can reduce long-run economic growth by limiting the size of social networks.

19 There was insufficient hospital capacity in Hubei (and Wuhan in particular) in late January. Most patients in Wuhan were hospitalized and isolated around mid February with the completion of new hospitals, makeshift health facilities, and increased testing capacity. See Section 5.1 for details.

20 We should note that the summary of China’s policy responses here is not a comprehensive list. Other entities have also made efforts to help curtail the spread of COVID-19. For example, on January 27, the State Grid Corporation of China declared that it would continue supplying electricity to resident users even if payment was not received on time. School and universities were closed already because of Lunar New Year holidays.

21 According to Law of the People’s Republic of China on Prevention and Treatment of Infectious Diseases, class A infectious diseases only include plague and cholera.

22 For a list of quarantine measures, see 2020 Hubei lockdowns (https://en.wikipedia.org/w/index.php?title=2020_Hubei_lockdowns&oldid=946423465), last visited April 2, 2020.

23 Wenzhou, Zhengzhou, Hangzhou, Zhumadian, Ningbo, Harbin, Fuzhou

24 This restriction varies from 1 to 5 days across cities. In most cities, such restrictions are once every 2 days. “Closed management of communities” and “family outdoor restrictions” were mostly announced in city-level government documents. There are some cities in which only part of their counties declared to implement “closed management of communities” or “family outdoor restriction” policy. However, other counties in the same city may have quickly learned from them. Thus, as long as one county in a city has implemented “closed management of communities” or “family outdoor restrictions,” we treat the whole city as having the policy in place.

## References

[CR1] Adda J (2016). Economic activity and the spread of viral diseases: evidence from high frequency data. Q J Econ.

[CR2] Ahrens A, Hansen C, Schaffer ME (2019) lassopack: model selection and prediction with regularized regression in Stata, arXiv:1901.05397

[CR3] Barmby T, Larguem M (2009). Coughs and sneezes spread diseases: an empirical study of absenteeism and infectious illness. J Health Econ.

[CR4] Belloni A, Chernozhukov V, Hansen C, Kozbur D (2016). Inference in high-dimensional panel models with an application to gun control. J Bus Econ Stat.

[CR5] Chin Y-M, Wilson N (2018). Disease risk and fertility: evidence from the HIV/AIDS pandemic. J Popul Econ.

[CR6] Chinazzi M, Davis JT, Ajelli M, Gioannini C, Litvinova M, Merler S, Pastore y Piontti A, Mu K, Rossi L, Sun K, Viboud C, Xiong X, Yu H, Halloran ME, Longini IM, Vespignani A (2020) The effect of travel restrictions on the spread of the 2019 novel coronavirus (COVID-19) outbreak, Science 10.1126/science.aba9757 PMC7164386 32144116

[CR7] Dong Y, Mo X, Hu Y, Qi X, Jiang F, Jiang Z, Tong S (2020) Epidemiological characteristics of 2143 pediatric patients with 2019 coronavirus disease in China, Pediatrics

[CR8] Durevall D, Lindskog A (2011). Uncovering the impact of the HIV epidemic on fertility in Sub-Saharan Africa: the case of Malawi. J Popul Econ.

[CR9] Fang H, Wang L, Yang Y (2020) Human mobility restrictions and the spread of the novel coronavirus (2019-nCoV) in China. NBER Working Paper 26906 10.1016/j.jpubeco.2020.104272 PMC7833277 33518827

[CR10] Ferguson NM, Laydon D, Nedjati-Gilani G, Imai N, Ainslie K, Baguelin M, Bhatia S, Boonyasiri A, Cucunubá Z, Cuomo-Dannenburg G, Dighe A, Dorigatti I, Fu H, Gaythorpe K, Green W, Hamlet A, Hinsley W, Okell LC, van Elsland S, Thompson H, Verity R, Volz E, Wang H, Wang Y, Walker PG, Walters C, Winskill P, Whittaker C, Donnelly CA, Riley S, Ghani AC (2020) Impacts of non-pharmaceutical interventions (NPIs) to reduce COVID-19 mortality and healthcare demand

[CR11] Fogli A, Veldkamp L (forthcoming) Germs, social networks and growth, Review of Economic Studies

[CR12] Gilbert M, Pullano G, Pinotti F, Valdano E, Poletto C, Boëlle P-Y, D’Ortenzio E, Yazdanpanah Y, Eholie SP, Altmann M, Gutierrez B, Kraemer MUG, Colizza V (2020) Preparedness and vulnerability of African countries against importations of COVID-19: a modelling study, The Lancet 10.1016/S0140-6736(20)30411-6 PMC7159277 32087820

[CR13] Godzinski A, Suarez Castillo M (2019) Short-term health effects of public transport disruptions: air pollution and viral spread channels, Working paper

[CR14] Guan W-J, Ni Z-y, Hu Y, Liang W-h, Ou C-q, He J-x, Liu L, Shan H, Lei C-l, Hui DS, Du B, Li L-j, Zeng G, Yuen K-Y, Chen R-c, Tang C-l, Wang T, Chen P-y, Xiang J, Li S-y, Wang J-l, Liang Z-j, Peng Y-x, Wei L, Liu Y, Hu Y-h, Peng P, Wang J-m, Liu J-y, Chen Z, Li G, Zheng Z-j, Qiu S-q, Luo J, Ye C-j, Zhu S-y, Zhong N-s (2020) Clinical characteristics of 2019 novel coronavirus infection in China, medRxiv

[CR15] Li Q, Guan X, Wu P, Wang X, Zhou L, Tong Y, Ren R, Leung K, Lau EH, Wong JY, Xing X, Xiang N, Wu Y, Li C, Chen Q, Li D, Liu T, Zhao J, Liu M, Tu W, Chen C, Jin L, Yang R, Wang Q, Zhou S, Wang R, Liu H, Luo Y, Liu Y, Shao G, Li H, Tao Z, Yang Y, Deng Z, Liu B, Ma Z, Zhang Y, Shi G, Lam TT, Wu JT, Gao GF, Cowling BJ, Yang B, Leung GM, Feng Z (2020) Early transmission dynamics in Wuhan, China, of novel coronavirus-infected pneumonia. New England Journal of Medicine 10.1056/NEJMoa2001316 PMC7121484 31995857

[CR16] Litvinova M, Liu Q-H, Kulikov ES, Ajelli M (2019). Reactive school closure weakens the network of social interactions and reduces the spread of influenza. Proc Natl Acad Sci.

[CR17] Liu Y, Gayle AA, Wilder-Smith A, Rocklöv J (2020) The reproductive number of COVID-19 is higher compared to SARS coronavirus, Journal of Travel Medicine 10.1093/jtm/taaa021 PMC7074654 32052846

[CR18] Lowen AC, Steel J (2014). Roles of humidity and temperature in shaping influenza seasonality. J Virol.

[CR19] Markowitz S, Nesson E, Robinson J (2019). The effects of employment on influenza rates. Econ Hum Biol.

[CR20] Maurer J (2009). Who has a clue to preventing the flu? unravelling supply and demand effects on the take-up of influenza vaccinations. J Health Econ.

[CR21] Milusheva S (2017) Less bite for your buck: using cell phone data to target disease prevention, Working paper

[CR22] Mizumoto K, Kagaya K, Zarebski A, Chowell G (2020). Estimating the asymptomatic proportion of coronavirus disease 2019 (COVID-19) cases on board the diamond princess cruise ship, yokohama, japan, 2020. Eurosurveillance.

[CR23] National Health Commission of the PRC (2020) Novel coronavirus pneumonia diagnosis and treatment plan (provisional 6th edition)

[CR24] Nishiura H, Kobayashi T, Miyama T, Suzuki A, Jung S, Hayashi K, Kinoshita R, Yang Y, Yuan B, Akhmetzhanov AR et al (2020) Estimation of the asymptomatic ratio of novel coronavirus infections (COVID-19), medRxiv 10.1016/j.ijid.2020.03.020 PMC7270890 32179137

[CR25] Oster E (2012). Routes of infection: exports and HIV incidence in Sub-Saharan Africa. J Eur Econ Assoc.

[CR26] Pichler S, Ziebarth NR (2017). The pros and cons of sick pay schemes: testing for contagious presenteeism and noncontagious absenteeism behavior. J Public Econ.

[CR27] Puhani PA (2020) France and Germany exceed Italy, South Korea and Japan in temperature-adjusted Corona proliferation: a quick and dirty Sunday morning analysis, GLO Discussion Paper, No. 487

[CR28] Shen C, Taleb NN, Bar-Yam Y (2020) Review of Ferguson et al “Impact of non-pharmaceutical interventions.., New England Complex Systems Institute

[CR29] Slusky D, Zeckhauser RJ (2018) Sunlight and protection against influenza NBER Working Paper 24340 10.1016/j.ehb.2020.100942 33340885

[CR30] Tian H, Liu Y, Li Y, Wu C.-H, Chen B, Kraemer MUG, Li B, Cai J, Xu B, Yang Q, Wang B, Yang P, Cui Y, Song Y, Zheng P, Wang Q, Bjornstad ON, Yang R, Grenfell BT, Pybus OG, Dye C (2020) An investigation of transmission control measures during the first 50 days of the COVID-19 epidemic in China, Science 10.1126/science.abb6105 PMC7164389 32234804

[CR31] Wang C, Liu L, Hao X, Guo H, Wang Q, Huang J, He N, Yu H, Lin X, Pan A et al (2020a) Evolving epidemiology and impact of non-pharmaceutical interventions on the outbreak of coronavirus disease 2019 in Wuhan, China, medRxiv

[CR32] Wang M, Jiang A, Gong L, Luo L, Guo W, Li C, Zheng J, Li C, Yang B, Zeng J, Chen Y, Zheng K, Li H (2020b) Temperature significant change COVID-19 transmission in 429 cities, medRxiv

[CR33] White C (2019) Measuring social and externality benefits of influenza vaccination Journal of Human Resources

[CR34] World Health Organization (2020a) Novel coronavirus situation report 7

[CR35] World Health Organization (2020b) Report of the WHO-China joint mission on Coronavirus Disease 2019 (COVID-19)

[CR36] Wu JT, Leung K, Leung GM (2020a) Nowcasting and forecasting the potential domestic and international spread of the 2019-nCoV outbreak originating in Wuhan China: a modelling study, Lancet 10.1016/S0140-6736(20)30260-9 PMC7159271 32014114

[CR37] Wu K, Zheng J, Chen J (2020b) Utilize state transition matrix model to predict the novel Corona virus infection peak and patient distribution, medRxiv

[CR38] Wu Z, McGoogan JM (2020c) Characteristics of and important lessons from the coronavirus disease 2019 (COVID-19) outbreak in China: summary of a report of 72314 cases from the Chinese Center for Disease Control and Prevention, JAMA 10.1001/jama.2020.2648 32091533

[CR39] Zhan C, Tse C, Fu Y, Lai Z, Zhang H (2020) Modelling and prediction of the 2019 Coronavirus Disease spreading in China incorporating human migration data, SSRN 10.1371/journal.pone.0241171 PMC7591076 33108386

[CR40] Zhang P, Zhang J, Chen M (2017). Economic impacts of climate change on agriculture: the importance of additional climatic variables other than temperature and precipitation. J Environ Econ Manag.

[CR41] Zhang C, Chen C, Shen W, Tang F, Lei H, Xie Y, Cao Z, Tang K, Bai J, Xiao L, Xu Y, Song Y, Chen J, Guo Z, Guo Y, Wang X, Xu M, Zou H, Shu Y, Du X (2020) Impact of population movement on the spread of 2019-nCoV in China, SSRN 10.1080/22221751.2020.1760143 PMC7269026 32321369

[CR42] Zhao S, Lin Q, Ran J, Musa SS, Yang G, Wang W, Lou Y, Gao D, Yang L, He D et al (2020) Preliminary estimation of the basic reproduction number of novel coronavirus (2019-nCoV) in China, from 2019 to 2020: a data-driven analysis in the early phase of the outbreak. International Journal of Infectious Diseases 10.1016/j.ijid.2020.01.050 PMC7110798 32007643

[CR43] Zhu N, Zhang D, Wang W, Li X, Yang B, Song J, Zhao X, Huang B, Shi W, Lu R, Niu P, Zhan F, Ma X, Wang D, Xu W, Wu G, Gao GF, Tan W (2020). A novel coronavirus from patients with pneumonia in China, 2019. N Engl J Med.

